# Antimicrobial Efficiency of Essential Oil and Microemulsion of *Pectis brevipedunculata* (Asteraceae): Evaluation of Bacterial and Fungal Inhibition in Strawberries

**DOI:** 10.1021/acsomega.6c00168

**Published:** 2026-04-22

**Authors:** Auxiliadora C. C. B. Lopes, Roberto B. de Lima, Renato S. Gonçalves, Carlos Eduardo Lima de Oliveira, Glécilla C. de S. Nunes, Amanda M. Teles, Matheus O. do Nascimento, André L. M. Carvalho, Cláudia Q. da Rocha

**Affiliations:** † Graduate Program in Chemistry, Federal University of Maranhão, São Luís, Maranhão 65080-805, Brazil; ‡ Department of Engineering and Exact Sciences, Setor Palotina, Federal University of Paraná, Palotina, Palotina 85950-000, Brazil; § Mechanical Engineering Department, 42487 State University of Maringá, Maringá, Palotina 87020-900, Brazil; ∥ Professional Postgraduate Program in Animal Health Defense, State University of Maranhão, São Luís, Maranhão 65055-310, Brazil; ⊥ Graduate Program in Pharmaceutical Sciences, Federal University of Piauí, Teresina, Piauí 64049-550, Brazil

## Abstract

*Pectis brevipedunculata*, commonly known as field rosemary, belongs to the Asteraceae family. This species’ leaves contain citral among their major compounds, a chemical marker of their essential oils (EO), among their major compounds. EO from *Pectis* species, which has antimicrobial, nematicidal, and larvicidal effects. The species is widely distributed throughout Brazil and, although it is popularly used in the form of tea, it has no significant use. This study aimed to obtain essential oil (EO) from the aerial parts of *P. brevipedunculata* (EO-PB), develop a microemulsion (ME) for incorporating the EO (ME-PB), and evaluate its antimicrobial potential when applied to strawberries. Chemical characterization by GC/MS of the hydrodistilled essential oil (EO) revealed that its main EO components were α-pinene (56.47%), limonene (19.98%), neral (3.78%), and geranial (3.46%). Analysis of the ME by GC/MS-Headspace and infrared spectroscopy confirmed the incorporation of the EO into the formulation. Physical-chemical characterization showed that the particle size, polydispersity index and zeta potential were consistent with published data. Regarding antimicrobial potential, the ME-PB formulation exhibited inhibition halos exceeding 50.00 mm against all tested bacteria (*Escherichia coli* (ATCC 25922), *Salmonella* spp. (ATCC 14028), *Listeria monocytogenes* (ATCC 35152), *Staphylococcus aureus* (ATCC 25923), *Bacillus cereus* (ATCC 14579) and *Clostridium* spp.). Notably, the inhibition halos were larger compared to those of the antibiotics penicillin and gentamicin. In contrast, EO-PB exhibited smaller inhibition halos for all the tested bacteria, except for *S. aureus*. It is noteworthy that antibiotics and essential oils exhibit different diffusion behaviors in agar. ME-PB indicated minimum inhibitory concentration values lower than those obtained by the action of the EO, and was considered to have strong inhibitory properties against all the bacteria tested. When EO and ME were applied to strawberries, it was found that EO-PB and ME-PB were effective against mesophilic aerobes and fungi, inhibiting the proliferation of microorganisms and extending the shelf life of strawberries. The results demonstrate the species’ potential and show that incorporating EO into ME optimizes the bioavailability of bioactive principles and improves biological activity.

## Graphical abstract

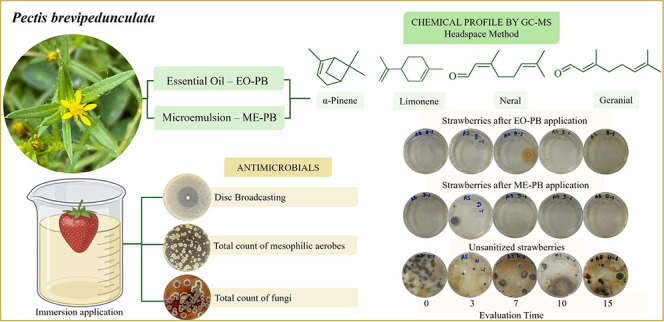

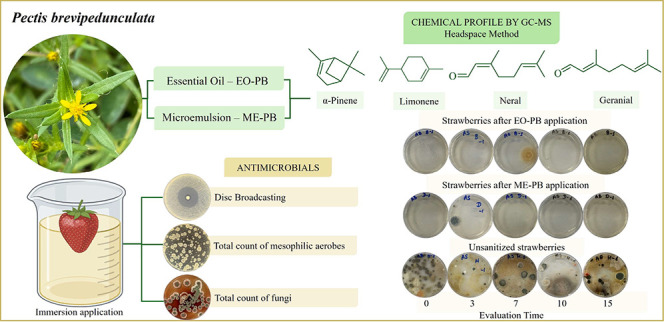

## 1 Introduction

The Brazilian flora is recognized for its exceptional biodiversity and distribution across multiple biomes. It includes distinct varieties of plants that serve as sources of chemically and biotechnologically relevant natural products.

*Pectis brevipedunculata* (Gardner) Sch. Bip. belongs to the Asteraceae Family and the *Pectis* L. genus, is commonly known as field rosemary, lemongrass, ant catinga, little lemon and lemongrass.^1–3^ *P. brevipedunculata* is a herbaceous plant endemic to Brazil. It is distributed across in the caatinga and cerrado ecosystems and is found perennially in several Brazilian states, including Pará, Maranhão, Piauí, Ceará, Pernambuco, Bahia, Goiás, Brasília, Minas Gerais, and Rio de Janeiro.^4^ This small aromatic species measures between 2 and 26 cm in height. It is adapted to xerophytic environments and is often found growing spontaneously in lawns and uncultivated fields. It is considered an ornamental aromatic grass.^4–6^

This species’ leaves contain citral, a mixture of the isomeric oxygenated monoterpenes neral and geranial among their major compounds. Citral is considered a chemical marker of the essential oils (EOs) of several *Pectis* species of the genus *Pectis*, such as *Pectis apodocephala*, *P. angustifolia*, *P. brevipedunculata*, *P. elongata*, and *P. linifolia*.^6^ In *P. brevipedunculata*, citral is associated with its calming effects, is well as the traditional use of the aerial parts to make teas.^7–9^ The literature reports other traditional uses of citral for species of the genus *Pectis*, including the treatment of anxiety, hypertension and intestinal problems, as well as its use as a flavouring agent, and as a remedy for colds and fever.^6^

These ethnopharmacological properties emphasize the biological significance of citral and justify the exploration of *P. brevipedunculata* essential oil for biotechnological applications. Citral exhibits cardiovascular effects such as transient hypotension and bradycardia,^10^ effects on the central nervous system of rats,^11^ vasodilatory activity in the isolated thoracic aorta of rats,^12^ potential as a insecticide for controllling of *Drosophila suzukii* (Matsumura), selectivity for nontarget organisms such as the honeybees *Apis mellifera* (Linnaeus) and *Partamona helleri* (Friese),^13^ citral also performs biological activities, such as bacterial and fungal strains.^6^

Essential oils derived from species of the *Pectis* genus (*P. apodocephala* and *P. oligocephala*) have potential nematicidal and larvicidal properties, exhibiting activity against *Meloidogyne incognita* and *Aedes aegypti*.^14^ These findings reinforce the biotechnological relevance of *P. brevipedunculata* and support its exploration in chemicals, biology, pharmacology, and food-related applications. The Research Group of the Laboratory of Chemistry of Natural Products (LQPN) at the Federal University of Maranhão (UFMA), São Luís Bacanga Campus, is investigating the biotechnological potential of the plant species selected for this study, where different activities are observed.

Microemulsion technology has achieved promising results in optimizing biological activities by increasing the bioavailability of bioactive principles.^15^ Microemulsions (MEs) are isotropic, optically transparent nanometric systems formed from an aqueous phase, an oily phase, and surfactants or surface-active agents.^16^ These systems are particularly efficient at solubilizing lipophilic compounds, especially essential oils.^17^

Microemulsion formulations can be used, among other applications, to preserve and extend the shelf life of foods,^17^ such as strawberries, which have a fragile surface layer that can easily become damaged. Combined with their high sugar content, low pH, and ideal water activity, this makes them susceptible to the growth of microorganisms.^18,19^ These intrinsic characteristics of the fruit emphasize the importance of research into the development of new protective agents.

MEs have great potential in the food industry as they have been developed to optimize antimicrobial activity against bacteria and fungi, prevent lipid oxidation, and avoid changes in quality parameters, such as appearance, firmness, and color resulting from deterioration or enzymatic reactions.^15^ Thus, the importance of ME-based systems for food preservation is clear.

To the best of our knowledge, there are no reports in the literature on the use ME with the incorporation of *P. brevipedunculata* EO for food purposes. Therefore, this study is innovative, as it explores a native natural from the Maranhão flora with previously uninvestigated technological applications. This study aimed to chemically characterize the essential oil (EO) obtained from *P. brevipedunculata* (EO-PB) and the ME formulated with the incorporation of this oil (ME-PB), as well as to assess the biotechnological potential of the species by evaluating the antimicrobial activity of both systems against mesophilic aerobes and fungi present in strawberries.

## 2 Results and Discussion

### 2.1 Chemical Characterization of Essential Oil and Microemulsion

The essential oil (EO-PB) extracted from the aerial parts of *P. brevipedunculata* yielded 0.67% and contained the following major compounds: α-pinene (56.47%) and limonene (19.98%) as the major compounds, followed by neral (3.78%) and geranial (3.46%). In the microemulsion (ME-PB) formulated with this essential oil (EO), the compounds present in the highest concentrations were α-pinene (50.17%), limonene (23.17%), neral (4.22%), and geranial (3.90%).^20^ All the volatile compounds identified in the EO-PB using the GC/MS method, as well as the compounds analyzed in the ME-PB using the GC/MS-headspace method are described in Lopes et al.,^20^ where the initial results obtained from the research are published, such as the chemical characterization of the EO, the development and characterization of the ME.

The presence of the major constituents of EO-PB in ME-PB indicates the successful incorporation of the essential oil and the maintenance of its active principles within the formulation. Compounds corresponding to 52% of the total ME composition are also present in the EO that was added to the formulation. The presence of these substances in the formulation suggests that EO-PB has been incorporated into the ME-PB system, preserving the active ingredients and enhancing biological activity.

The characterization of EO-PB and ME-PB was also performed using infrared (IV) spectroscopy. The analysis revealed the presence of the following bands in EO-PB: 2968 cm^–1^, representing C–H stretching; 1674 cm^–1^, corresponding to C=C stretching; 1377 cm^–1^, which corresponds to the average symmetric deformation in CH_3_; and the 842 cm^–1^ cm^–1^ (aromatic ring deformation) according to Donald et al.^21^ These same bands were also observed in ME-PB, reinforcing the successful incorporation of EO into the microemulsion matrix. Complementing these bands, similar regions were also observed at 2916 cm^–1^ and 2922 cm^–1^ (C–H stretching); 1720 cm^–1^ and 1735 cm^–1^ (C=O stretching); 1444 cm^–1^ and 1454 cm^–1^ (asymmetric CH_3_ deformation), present in EO-PB and ME-PB, respectively (Figure 1).

**Figure 1:**
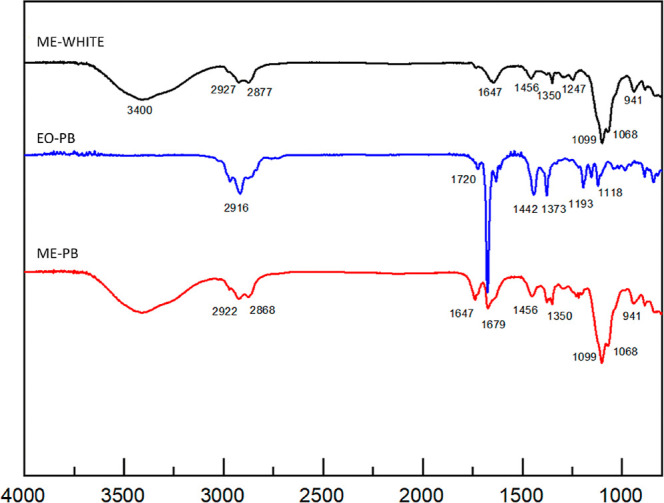
Spectra obtained in the infrared region for the essential oil (EO-PB), the microemulsion (ME-PB) of *Pectis brevipedunculata*, and its blank (ME-WHITE).

In the EO, it was also possible to verify some bands, 1193 cm^–1^ and 1120 cm^–1^, of C=O stretching, which were not visualized in the ME spectrum, possibly due to the overlap of the bands in this region (Figure 1).

In ME-PB, some other frequencies were verified, which are also present in the spectrum of the white microemulsion, i.e., formulation developed with surfactants and distilled water, without the addition of essential oil (ME-WHITE), originating from the surfactants used in the formulation, which are 1350 cm^–1^; 1099 cm^–1^; 1068 cm^–1^ and 941 cm^–1^, representing C–O deformation and angular deformation outside the C=C plane (Figure 1).

ME-PB and ME-WHITE were characterized by IV before and after the ME thermal stability test, to observe whether temperature variation is responsible for changes in the chemical composition of the formulation (Figure 2). Only the 2968 cm^–1^ C–H stretching band was not observed after heating, likely due to reduced intensity or overlapping.

**Figure 2:**
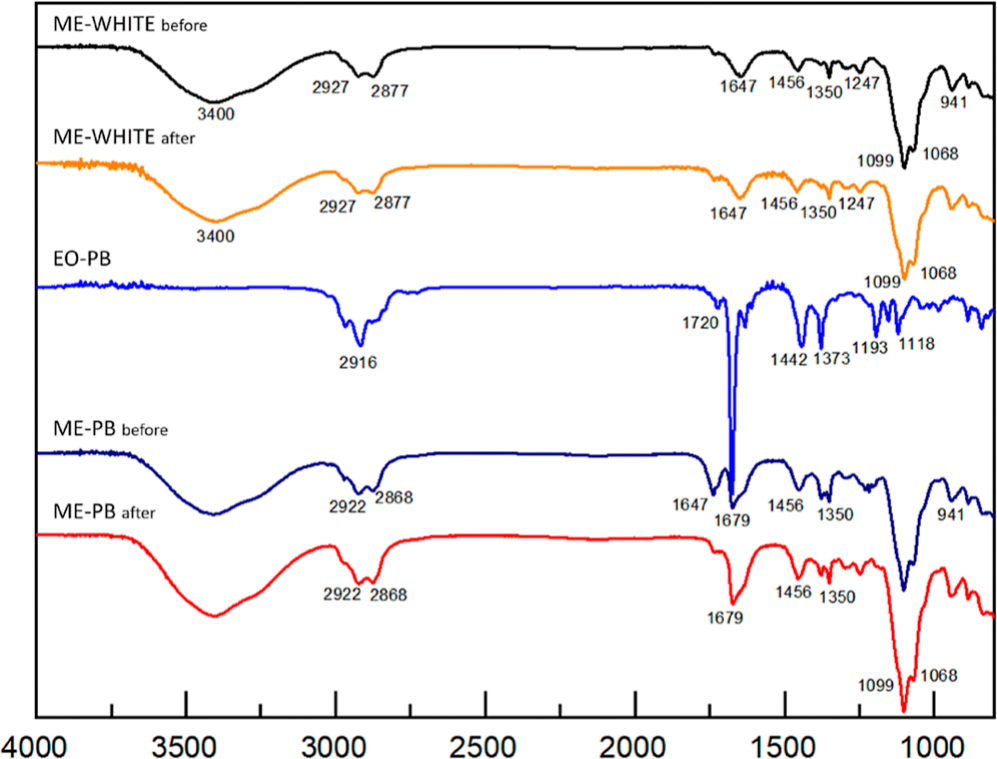
Spectra of the essential oil (EO-PB), microemulsion (ME-PB) of *Pectis brevipedunculata*, and its blank (ME-WHITE) before and after the thermal stability test of the microemulsion.

The thermal stability test proved that the temperature variation did not interfere with the composition of the ME vehicle, i.e., all bands of the surfactant spectrum (ME-WHITE) were preserved (Figure 2).

Similarly, in ME-PB, all bands were maintained before and after the thermal stability test, except the band present at 2968 cm^–1^, which corresponds to C–H stretching according to Donald et al.,^21^ and is signaled in the EO-PB and ME-PB spectrum before the test, but is not observed in the spectrum after the test (Figure 2).

As can be seen from Figure 2, the spectra obtained show that the chemical composition of the ME remained similar to its initial state after the temperature variation test, as most of the wavelengths were repeated.

### 2.2 Development of Microemulsion and Incorporation of Essential Oil

The pseudoternary phase diagram, from which the ME-PB proportions were extracted, was constructed by Lopes et al.^20^ From different proportions of surfactants/oil/water, 81 formulations were obtained, which gave rise to specific regions in the phase diagram, which are the regions of the Winsor Classification: WI (Winsor I, a two-phase system formed by an oil phase in equilibrium with an emulsified phase); WII (Winsor II, a two-phase system consisting of an aqueous phase in equilibrium with an emulsified phase); WIII (Winsor III, a three-phase system consisting of an aqueous phase and an oil phase, mediated by an emulsified phase); WIV (Winsor IV, a single-phase system);^22^ in addition to a milky white liquid emulsion and a cloudy system. In the WIV region of the phase diagram, there are single-phase, liquid, optically transparent, and stable systems, which are defined as microemulsions. From this region of microemulsions (MEs), the WIV region of the diagram, 18 MEs were obtained with different surfactant/oil/water ratios, with 9 MEs from formulations with a 9:1 ratio, 5 MEs with an 8:2 ratio, and 4 MEs with a 7:3 ratio. These ME formulations presented essential oil (EO) percentages ranging from 3.6 to 25%. The selected ME ratio was 7:3:4, which contains the EO incorporated into the system at a concentration of 20%, as explained by Lopes et al.^20^ This intermediate EO concentration was chosen considering the best utilization of the active ingredients and the best efficiency in the development of biological activities. The selected ME presented properties expected for this type of formulation, which are optical transparency, thermodynamic stability, and high EO solubilization capacity, making the active ingredients more bioavailable.

### 2.3 Physical-Chemical Characterization of Microemulsion

The physical-chemical characterization of ME-PB was performed by Lopes et al.^20^ The study determined that ME-PB is of the oil–water type, consisting of oil droplets dispersed in water, forming a transparent, clear, and homogeneous system. This morphological profile is consistent with classical microemulsion systems designed for the solubilization of lipophilic compounds. In the study, they concluded that the particle size (64.75 nm), polydispersity index (0.37), and zeta potential (−12.9 mV) of ME-PB and ME-WHITE (17.18 nm; 0.35; and −25 mV, respectively) are in accordance with what is reported in the literature for stable microemulsions. Zeta potential is associated with the surface potential of essential oil particles or surfactant charges. This parameter can have a positive or negative value, depending on the nature of the microemulsion components (oil and surfactants).^20^ Negative zeta potentials can be attributed to the presence of chemical groups in surfactants that ionize, forming negatively charged particles. Therefore, microemulsions formulated with nonionic surfactants, such as Tween 80, used in this study, can be stable even if they exhibit negative zeta potentials.^63^

After the thermal stability test, no significant variations were observed in density, demonstrating that temperature fluctuations did not compromise the structural integrity of the formulations. Lopes et al.^20^ inferred that after the temperature variation that occurred in the ME thermal stability test, there was no significant variation in density, ME-PB decreased from 0.99 to 0.98 g/mL, and ME-WHITE increased from 1.00 to 1.02 g/mL, both values remained within the expected range for microemulsion systems and did not indicate phase separation or destabilization. Regarding pH, after the stability test, there was a reduction in hydrogen ion potential in ME-PB from 7.09 to 5.46 and in ME-WHITE from 6.81 to 6.46. Even with this decrease, the pH of both systems remained within the suitable range (5.5–8.0) for physical-chemical stability, as reported in the literature. The researchers note that despite the reduction in pH, the values for this parameter are still within the range of greater physical-chemical stability for formulations, 5.5 to 8.0.^20^

In the present study, a rheological analysis of the microemulsion (ME) was performed. This determines whether the fluid’s behavior is related to the type and degree of organization of the microemulsion system.^23^ Both ME-PB and ME-WHITE displayed Newtonian behavior, characterized by constant viscosity and a linear relationship between shear stress and shear rate. This type of behavior stems from small particles present in the system that have low interaction between them.^24^

The rheograms (Figure 3) show overlapping ascending and descending curves, resulting in a straight line passing through the origin, confirming Newtonian flow. This pattern indicates that shear does not promote changes in microstructural arrangement, as expected for kinetically stable and low-viscosity microemulsions. Similar observations have been previously reported for microemulsion systems of comparable composition.^25–27^

**Figure 3:**
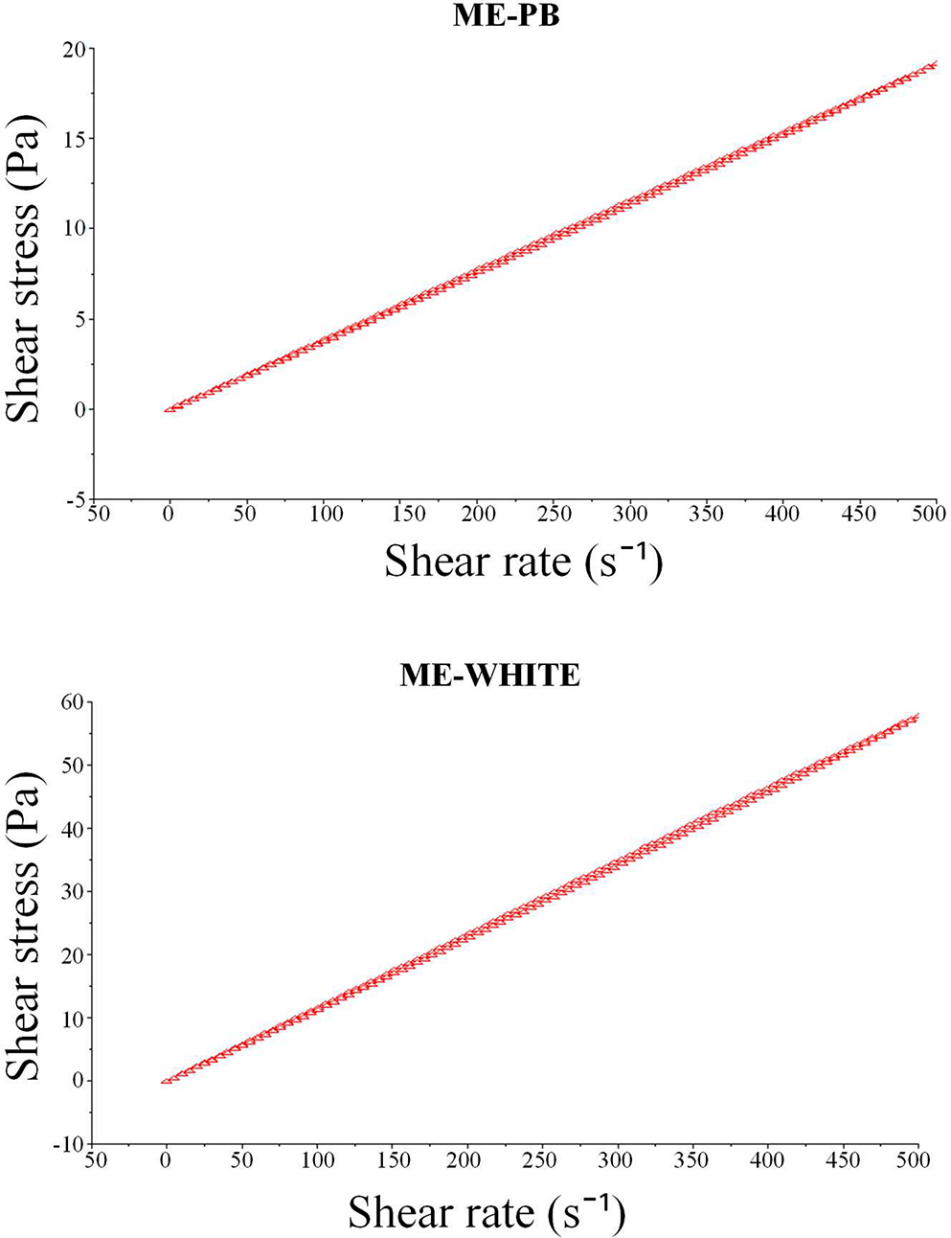
Reograms showing the relationship between the rate and shear stress of the microemulsion of *Pectis brevipedunculata* essential oil (ME-PB) and the microemulsion blank (ME-WHITE).

The Newtonian flow profile and the stability of viscosity across varying shear rates confirm the robustness and uniformity of the ME-PB system, reinforcing its applicability as a vehicle for controlled delivery of essential oil constituents. These rheological characteristics play a crucial role in determining handling properties, spreadability, and mechanical behavior of formulations intended for food preservation or technological applications.

In the generated line, all pairs of shear rate and stress values are constant. Therefore, the viscosity of Newtonian fluids is also constant, since this property is unaffected by changes in shear rate.^25^

These rheological characteristics determine the mechanical properties, physical stability and consistency of fluids and microemulsions.^27^

### 2.4 Evaluation of the Antimicrobial Activity of Essential Oil and Microemulsion

The antimicrobial action of EO-PB, ME-PB, and ME-WHITE was initially verified using the disk diffusion method, which analyzed the diameters of the inhibition halos produced. Similarly, the halos generated by the antibiotics gentamicin and penicillin were verified. Table 1 presents the mean measurement values, in mm, and standard deviations.

**Table 1:** Results of Antimicrobial Activity Using the Disk Diffusion Method for the Essential Oil (EO-PB) and Microemulsion (ME-PB) of *Pectis brevipedunculata* and Its Blank (ME-WHITE)^a^

	samples (mm)			antibiotics (mm)	
bacteria	EO-PB	ME-PB	ME-WHITE	gentamicin	penicillin
*Escherichia coli*	41.33 ± 7.57	>50.00 ± 0	>50.00 ± 0	27.33 ± 1.15	22.67 ± 1.15
*Staphylococcus aureus*	>50.00 ± 0	>50.00 ± 0	0	26.67 ± 1.15	38.00 ± 0
*Salmonela* spp.	23.67 ± 1.16	>50.00 ± 0	>50.00 ± 0	23.00 ± 1.00	19.00 ± 1.00
*Listeria monocytogenes*	32.67 ± 2.31	>50.00 ± 0	>50.00 ± 0	18.00 ± 0	18.00 ± 1.00
*Bacillus cereus*	17.67 ± 0.58	>50.00 ± 0	>50.00 ± 0	31.00 ± 1.00	26.00 ± 3.00
*Clostridium* spp.	17.67 ± 0.58	>50.00 ± 0	0	29.00 ± 1.00	18.00 ± 0

a Note: Mean values of triplicate measurements ± standard deviation.

ME-PB produced inhibition halos greater than 50.0 mm for all tested bacteria, demonstrating markedly enhanced antimicrobial performance compared to EO-PB, which ranged from 17.67 to 41.33 mm, with the exception of *Staphylococcus aureus*, which also presented an inhibition halo >50.0 mm. The superior efficacy of ME-PB highlights the contribution of the microemulsified system to increasing the bioavailability and dispersion of the essential oil constituents.

The EO-PB showed larger inhibition halos for *Escherichia coli*, *Staphylococcus aureus*, *Salmonella* spp., and *Listeria monocytogenes* when compared to the positive control antibiotics. However, for *Bacillus cereus* and *Clostridium*, the antibiotics showed larger inhibition halos. It is worth noting that the diffusion of antibiotics and essential oils in discs occurs differently, as antibiotics are water-soluble, causing good diffusion of these compounds in the agar used, which is also water-soluble. In contrast, essential oil, being lipid-soluble, does not diffuse in the same way in the agar, and may present compounds retained in the discs used and compounds volatilized. From this explanation of these different diffusion behaviors, it can be inferred that the results obtained for the essential oil do not signify a superior antimicrobial potency compared to the antibiotics tested (see Table 1).

The ME-WHITE formulated here did not present an inhibition halo for *S. aureus* and *Clostridium* spp., and as mentioned above, the EO showed a significant diameter halo, >50.0 mm (Table 1), this confirms that the antimicrobial effect of ME-PB originates from the active essential oil components and not from the surfactants. The halos observed for other bacteria in ME-WHITE may be related to the intrinsic lipophilic interactions of the surfactants with microbial cell membranes, potentially altering membrane permeability.

The other inhibition halos produced by ME-WHITE can be explained by the lipophilic nature of the substances present, which interfere with and modify the plasma membrane of these microorganisms, causing cell disruption.

The antimicrobial action of surfactants has been investigated for decades,^98,99^ and it has been proven that interaction with the cell membrane of bacteria occurs even at low concentrations, thus exerting a bactericidal and bacteriostatic effect.

Studies^97^ demonstrate that polysorbate-type surfactants (Tweens), when in low concentrations, their molecules intercalate into the cell membrane and thus modify membrane permeability, and when in high concentrations, solubilization causes cell rupture. Kaur and Mehta (2017)^97^ add that cell membrane permeability is facilitated when the formulation has high concentrations of surfactants, as the principle of Fick’s first law of diffusion occurs, which states that the diffusion flux is directly proportional to the concentration gradient, which may justify the results found for the microemulsion blank in the disk diffusion test, given that the formulation concentration of 200 mg/mL, and the percentage of surfactants (in a 1:1 ratio) of approximately 26.7%, are high, which may have generated a significant diffusion of the microemulsion in the agar culture medium promoting inhibition halos >50.0 mm.

It should be added that the microemulsion developed in this research is of the oil-in-water type, that is, it consists of oil droplets dispersed in water. Thus, the predominant phase in the formulation is aqueous. Similarly, the Mueller Hinton agar (Merck, Darmstadt, Germany) used in the disk diffusion test is water-soluble, which allows for good diffusion of the components of the microemulsion system in the medium.

Smaller inhibition zone diameters were produced by the EO of *P. brevipedunculata*, as researched by Marques et al.,^6^ who found 10 mm for *E. coli* and 22 mm for *S. aureus*. The lower efficacy reported previously may be explained by differences in solubilization methods, particularly the dilution of essential oil (EO) in dimethyl sulfoxide (DMSO) in the previous study. These results were obtained for both Gram-negative and Gram-positive bacteria, which may justify the results found.

The compounds that stand out for performing the antimicrobial activity of *P. brevipedunculata* are the isomers Z-citral or neral and E-citral or geranial, which constitute citral, a monoterpene that occurs naturally in herbs, plants, and citrus fruits. It has antifungal, bactericidal, insecticidal, and expectorant activity, has spasmolytic properties, and also has diuretic and anti-inflammatory effects.^28^ This monoterpene possesses antifungal, bactericidal, insecticidal, spasmolytic, and anti-inflammatory properties, and its mechanism of action involves membrane disruption, protein denaturation, and increased permeability leading to cell lysis.

A study was conducted to verify the bactericidal activity of a citral-based nanoemulsion, which produced inhibition halos measuring 9.4 mm for *E. coli*, 19.2 mm for *S. aureus*, and 14.4 mm for *L. monocytogenes*.^28^ These results were all lower than those found for ME-PB in the present study. This further highlights the effectiveness of the ME-PB system in enhancing antimicrobial delivery.

Other monoterpene compounds found in *P. brevipedunculata*, such as α-pinene and limonene, have also been shown to have antimicrobial properties.^29^

The minimum inhibitory concentrations (MICs), in μg/mL, of EO-PB, ME-PB, ME-WHITE, and of the antibiotics ampicillin and gentamicin against bacterial strains, using the broth dilution method, are shown in Table 2.

**Table 2:** Results of Antimicrobial Activity Using the Broth Dilution Method, Determining the Minimum Inhibitory Concentration (MIC) of the Essential Oil (EO-PB) and Microemulsion (ME-PB) of *Pectis brevipedunculata* and Its Blank (ME-WHITE)^a^

	samples (μg/mL)			A antibiotics	
bacteria	EO-PB	ME-PB	ME-WHITE	ampicillin	gentamicin
*Escherichia coli*	125.00	62.50	>1600	18.00	8.00
*Staphylococcus aureus*	125.00	62.50	>1600	10.00	6.00
*Salmonela* spp.	1000	250.00	>1600	16.00	8.00
*Listeria monocytogenes*	1000	62.50	>1600	24.00	18.00
*Bacillus cereus*	250.00	125.00	>1600	20.00	16.00
*Clostridium* spp.	1200	250.00	>1600	16.00	16.00

a Source: Authors.

EO-PB exhibits strong antimicrobial inhibition against *E. coli*, *S. aureus*, and *B. cereus*, with an MIC ≤ 500 μg/mL (see Table 2). It exhibits moderate antimicrobial inhibition against *Salmonella* spp., *L. monocytogenes*, and *Clostridium* spp., with an MIC ranging from 600 to 1500 μg/mL, in accordance with the recommendations of Aligiannis et al.^31^

ME-PB showed significantly lower MIC values (62.50–250.00 μg/mL), indicating strong inhibition for all bacteria evaluated and confirming the enhanced antimicrobial potency of the microemulsion system.

ME-WHITE, as already mentioned in the previous section, is considered, according to the guidelines of Ayres et al.,^32^ an inactive sample, with MIC > 1600 μg/mL (Table 2).

Other species of the genus *Pectis* that contain geranial and neral as major components also exhibit bactericidal activity, such as *P. elongata* Kunth., which obtained MIC values of 15.10 μg/mL for *L. monocytogenes* and 29.20 μg/mL for *B. cereus*.^33^

The bioactive compounds present in the essential oil of *P. brevipedunculata*, mainly terpenes, α-pinene (56.47%), limonene (19.98%), and citral, composed of neral (3.78%) and geranial (3.46%), which were incorporated into the microemulsion, inhibit microorganisms through mechanisms of action that involve the modification of the bacterial cell wall, promoting intrinsic effects such as protein denaturation, loss of cytoplasmic material, and resulting in cell death.^28,29^

Limonene has lipophilic characteristics, and the solubilization of lipids in the plasma membrane can lead to cell lysis. In its mechanisms of action in bacteria, limonene increases cell membrane permeability, alters membrane potential, decreases heat resistance, and causes membrane fluidization. It also significantly alters cytoplasmic homogeneity and cell wall thickness. Furthermore, in bacteria, limonene causes the leakage of substances from inside the cells, such as proteins, lipids, and nucleic acids; it decreases ATP concentration; it inhibits the synthesis of proteins in the respiratory chain complex, decreasing respiratory chain activity and interfering with respiratory function and energy metabolism; and it modifies DNA conformation, affecting cellular stability and potentially leading to cell degradation and death.^30^

Citral penetrates the lipid structure of bacterial cell walls, denatures proteins, and generates cytoplasmic leakage.^28^ Citral affects the bacterial cell membrane, causing hyperpolarization, in addition to other damage that modifies its integrity. Furthermore, it alters the fatty acid composition of the membrane, inhibits enzymes of microbial metabolism, reduces pH and intracellular ATP concentration, interferes with intercellular communication (quorum sensing), decreases extracellular polysaccharide production, alters the transcription of genes involved in hemolysin production, and reduces biofilm formation. Depending on the concentration of citral, it may present different targets in its mechanism of action in bacteria. Being a monoterpene aldehyde, citral should have a mechanism of action similar to that of other aldehydes. When this compound is present in low concentrations, it can promote the cross-linking of amino groups in the cell wall and cytoplasm, and also inhibit enzymes in the cytoplasmic membrane. Even at high concentrations, citral can affect the cytoplasm, causing coagulation and precipitation of its components.^100^

α-Pinene significantly modifies the cell membrane of bacteria, affects extracellular proteins and polysaccharides, and reduces the expression of key genes in biofilm formation and maturation.^101^

When these compounds are incorporated into formulations, the bioactive release system occurs through four main delivery routes: passive transport across the outer cell membrane enhances interaction with the cytoplasmic membrane; surfactant particles interact with the phospholipid bilayer of the cell membrane and promote targeted release of the essential oil; controlled release of essential oil droplets occurs, partitioned between the oil phase and the aqueous phase of the formulation, thus prolonging the activity of the compounds; and the concentration of essential oil at the site of action increases due to the electrostatic interaction of positively charged formulation droplets with negatively charged microbial cell walls.^28^

Despite the proven biological activities of limonene and citral, these compounds exhibit certain characteristics that necessitate strategies for better utilization. Limonene possesses hydrophobic and oxidative degradation properties, volatilizes under light, air, humidity, and high temperature, and is sensitive to oxidation and chemical transformation. Citral is insoluble in water at neutral pH and its antimicrobial activity may be reduced due to its susceptibility to oxidative degradation under normal storage conditions. Therefore, microemulsion technology can be employed to microencapsulate, solubilize, and protect these compounds, in order to then use them to inhibit microorganisms.^28,30^

To investigate the permanence of the effect of the active ingredients and biological activity of ME, the formulation was monitored before and after a thermal stability test. The same was done for the ME blank. Table 3 shows the results of antimicrobial activity using the broth dilution method, determining the minimum inhibitory concentration (MIC), in μg/mL, of ME-PB and ME-WHITE before and after the thermal stability test. The table highlights the statistical difference between the means of each sample, with a significance level of *p* ≤ 0.05.

**Table 3:** Results of Antimicrobial Activity Using the Broth Dilution Method, Determining the Minimum Inhibitory Concentration (MIC) of the Microemulsion (ME-PB) of *Pectis brevipedunculata* and Its Blank (ME-WHITE) before and after Thermal Stability Testing^a^ ^,^ ^b^

samples (μg/mL)				
bacteria	ME-PB before	ME-PB after	ME-WHITE before	ME-WHITE after
*Escherichia coli*	62.50^a^	125.00^b^	>1600^a^	>1600^a^
*Staphylococcus aureus*	62.50^a^	250.00^b^	>1600^a^	>1600^a^
*Salmonela* spp.	250.00^a^	125.00^b^	>1600^a^	>1600^a^
*Listeria monocytogenes*	62.50^a^	250.00^b^	>1600^a^	>1600^a^
*Bacillus cereus*	125.00^a^	125.00^a^	>1600^a^	>1600^a^
*Clostridium* spp.	250.00^a^	250.00^a^	>1600^a^	>1600^a^

a Source: Authors.

b Note: The statistical difference between the means of each sample was determined by the two-way ANOVA test with Tukey’s comparison (*p* ≤ 0.05), checking the parameters before and after the thermal stability test.

After the stability test, the antimicrobial activity of ME-PB was not affected, and the sample continued to show strong bacterial inhibition for all bacteria, with MICs of 125.00 and 250.00 μg/mL. Statistically comparing before and after the test, for *B. cereus* and *Clostridium* spp. bacteria there was no significant difference, for the others there was a significant difference, but as mentioned, it did not interfere with the antibacterial action.

ME-WHITE achieved MIC values >1600 μg/mL, demonstrating its inactivity. For all bacteria tested with ME white, there was no significant difference.

### 2.5 Application of Essential Oil and Microemulsion on Strawberries

The samples (EO-PB, ME-PB, and ME-WHITE) were applied to the strawberries, and then the pH, soluble solids content in °Brix, and moisture content were checked at time 0 and 15 days after immersion in the samples. Table 4 shows the mean values and standard deviations of these analyses. Initially, the unsanitized strawberry had a pH of 3.24, characterizing it as acidic. The fruit that was not sanitized with a 200 ppm sodium hypochlorite solution maintained its hydrogen ion potential throughout the 15 day evaluation period. The sanitized strawberries and those immersed in EO and ME of *P. brevipedunculata* showed higher pH values after 15 days compared to the unsanitized control group, but remained in the acidic pH range. The pH of strawberry pulp, considered the edible part of the fruit, must be equal to 3.3, according to Normative Instruction N°37 of October 1, 2018, issued by MAPA (Ministry of Agriculture, Livestock and Supply),^34^ and the pH of the edible portion of strawberries in their normal and natural state can still be in the range between 3.0 and 3.90, according to the Food and Drugs Administration (FDA).^35^ Therefore, the application of EO-PB and ME-PB on strawberries did not change the expected pH for the fruit.

**Table 4:** pH, Total Soluble Solids Content, and Moisture Content of Unsanitized Strawberries, Sanitized Strawberries, and Strawberries after Application of Essential Oil (EO-PB) and Microemulsion (ME-PB) of *Pectis brevipedunculata*, and Its Blank (ME-WHITE)^a^ ^,^ ^b^

time	samples	pH	°Brix	moisture
0 day	unsanitized strawberries	3.24 ± 0.01	5.30 ± 0	90.85% ± 0.02
15 days	unsanitized strawberries	3.24 ± 0.03	5.00 ± 0.01	89.18% ± 0.03
	sanitized strawberries	3.47 ± 0.01	5.00 ± 0.01	89.49% ± 0.03
	EO-PB	3.58 ± 0.02	6.30 ± 0.02	88.56% ± 0.01
	ME-PB	3.56 ± 0.02	10.20 ± 0.02	90.60% ± 0.01
	ME-WHITE	3.71 ± 0.01	10.20 ± 0.01	88.85% ± 0.02

a Source: Authors.

b Note: Mean values of triplicate measurements ± standard deviation.

The increase in pH in coated strawberries was also verified by Duran et al.^36^ In a study using chitosan, natamycin, nisin, pomegranate, and grape seed extract coatings, the researchers found pH levels ranging from 3.33 to 3.56 in uncoated strawberries and pH levels ranging from 3.33 to 3.49 in coated strawberries. They attribute the increase in pH to the use of organic acids during respiration during storage.

Regarding soluble solids content, the unsanitized strawberries analyzed at time 0 had 5.30 °Brix, and at 15 days, they had 5.00 °Brix. At the last observation time, the sanitized strawberries also showed 5.00 °Brix, and the groups of strawberries that were immersed in EO-PB, ME-PB, and ME-WHITE showed higher values of total soluble solids, of 6.30; 10.20, and 10.20°Brix, respectively (Table 4). The expected soluble solids content for strawberry pulp, according to IN N°37 of MAPA,^34^ is 6.50 °Brix. Thus, of the values obtained, those furthest from this reference value were those obtained with the application of ME-PB and ME-WHITE.

In the study by Duran et al.,^36^ it was also noted that the control group showed a reduction in total solids content during storage, from 10.86° to 8.22 °Brix. The reduction in this parameter was greater in the control group than in the other groups with coatings. Based on Aday and Caner,^37^ Duran et al.^36^ explain that in uncoated fruits, sucrose hydrolysis, which occurs in order to maintain physiological activity, developed at a faster rate than in the coated strawberry groups, resulting in a decrease in soluble solids. Duran et al.^36^ also found that during the first 5 days of storage, strawberries coated with chitosan and pomegranate extract showed an increase in soluble solids content, from 10.86° to 10.94 °Brix, as in the fruits treated with EO-PB, ME-PB, and ME-WHITE in this study.

Before the start of the procedure (time 0), the unsanitized strawberries had a moisture content of 90.85%. This moisture content is close to that cited in the Brazilian Food Composition Table, which is 91.50%.^38^ After 15 days, moisture decreased in all treatment groups, except for the group treated with ME-PB, which had a moisture content close to that at the start of the experiment, at 90.60%. Of the other groups, those with the lowest moisture content were those treated with EO-PB and ME-WHITE, with 88.56% and 88.85%, respectively (Table 4).

The reduction in strawberry weight over the 15 day period was increasing. Table 5 presents the weight loss of strawberries after application of EO-PB, ME-PB, and ME-WHITE. Except for the 10 and 7 day periods for strawberries treated with EO-PB and ME-PB, respectively. Of the groups evaluated, the unsanitized strawberries and the strawberries treated with ME-WHITE lost more weight than the fruits treated with EO-PB and ME-PB, with weight loss rates rising from 0.14 to 4.32% and from 0.47 to 4.89%, respectively. The coatings on the fruit act as an extra layer, covering the stomata, causing a reduction in transpiration and, consequently, a reduction in weight loss,^39^ which was noted when comparing the ME-PB application group with the group of unsanitized strawberries.

**Table 5:** Weight Loss of Strawberries after Application of Essential Oil and *Pectis brevipedunculata* Microemulsion, and White Microemulsion^a^

	samples				
time	unsanitized strawberries (%)	sanitized strawberries (%)	EO-PB (%)	ME-PB (%)	ME-WHITE (%)
3 days	0.14	0.13	0.36	1.45	0.47
7 days	2.05	0.34	1.37	1.19	1.17
10 days	2.08	0.86	1.09	1.91	1.84
15 days	4.32	0.97	1.51	2.05	4.89

a Source: Authors.

In a study of strawberries coated with edible chitosan-based coatings, Khan et al.^19^ observed that as storage time increased, there was also an increase in weight loss in all strawberry treatment groups. The control group lost 2.02% of its weight, while the coated strawberry group lost 1.37% of its weight at the end of the 8 day storage period. Guerreiro et al.^39^ studied edible coatings based on polysaccharides, sodium alginate, and pectin, enriched with essential oil constituents, citral, and eugenol, and found that strawberry weight loss increased in all treatments during 14 days of storage at 0.5 °C. The control lost 4.60% after 14 days of storage. In coatings with various polysaccharide formulations, including different proportions of citral and eugenol, weight loss ranged from 4.00% to 9.90%.

The visual decay of strawberries was analyzed, and those with visible dark lesions, representing areas infected by microorganisms, as defined by Khan et al.,^19^ were classified as spoiled. In addition to this aspect, changes in texture were analyzed, including loss of firmness and the presence of fungal infection. Table 6 displays the evaluation of visual deterioration, apparent fungal infection, and rot rate of groups of strawberries after application of EO-PB, ME-PB, and ME-WHITE. It was noted that after 3 days of analysis, the group of unsanitized strawberries began to show damage to the fruit structure, notably a change to dark coloration and altered texture in these regions (Table 6 and Figure 4). The rate of decay for this first group during the analysis period was 50.00%. The group of strawberries that were sanitized with 200 ppm sodium hypochlorite showed the same characteristics as the group mentioned above; however, the deterioration was lower, at 16.67%. After 7 days, the changes intensified, with 75.0% of the unsanitized strawberries and 50.00% of the sanitized strawberries already considered deteriorated. After 10 days, visible fungal growth appeared in these groups, with 100.00% rot in strawberries that had not been sanitized and 66.67% rot in strawberries that had been treated with the sanitizer. On the last day of analysis, the latter group had a 83.33% rot rate (Table 6 and Figure 4).

**Table 6:** Evaluation of Visual Decay, Apparent Fungal Infection, and Rotting Rate of Strawberry Groups after Application of Essential Oil and Microemulsion of *Pectis brevipedunculata*, and Microemulsion Blank^a^

strawberry groups from the application	time 0	time 3 days	time 7 days	time 10 days	time 15 days
EO-PB	-	-	-	-	-
ME-PB	-	-	-	-	-
ME-WHITE	-	-	visible lesion; darkening; change in texture 66.67%	visible lesion; darkening; change in texture 66.67%	visible lesion; darkening; change in texture 100.00%
sanitized strawberry	-	visible lesion; darkening; change in texture 16.67%	visible lesion; darkening; change in texture 50.00%	visible lesion; darkening; change in texture; apparent fungal infection 66.67%	visible lesion; darkening; change in texture; apparent fungal infection 83.33%
unsanitized strawberries	-	visible lesion; darkening; change in texture 50.00%	visible lesion; darkening; change in texture 75.00%	visible lesion; darkening; change in texture; apparent fungal infection 100.00%	visible lesion; darkening; change in texture; apparent fungal infection 100.00%

a Source: Authors.

**Figure 4:**
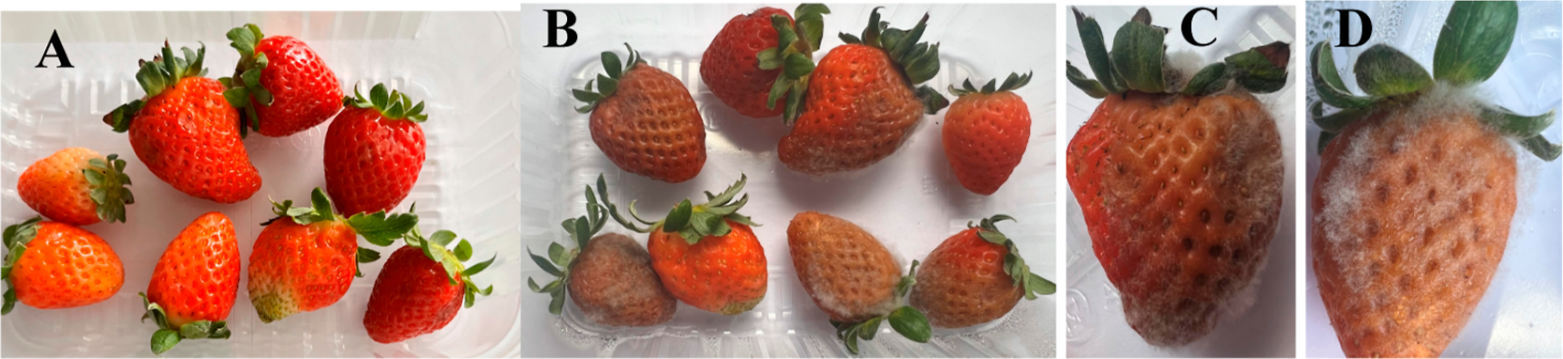
Group of strawberries on the day of sanitization with a 200 ppm sodium hypochlorite solution (A) and after 15 days (B), showing visual decay (C) and apparent fungal infection (D).

The strawberries treated with ME-WHITE began to show visible lesions, darkening, and loss of firmness within 7 days, with a deterioration rate of 66.67%. After 10 days, the rate remained the same, and after 15 days, it rose to 100.0% (Table 6).

The strawberries treated with EO-PB and ME-PB showed no structural changes, no signs of visual decay, and no apparent fungal growth (Table 6).

A study evaluating visible fungal infection in strawberries found that microorganisms proliferated after 4 days of storage at 10 °C. In the present study, we only verified the presence of fungi after 10 days. However, it should be noted that the storage conditions were different, with a storage temperature of 5 °C. Khan et al.^19^ infer that the storage temperature parameter affects the quality and shelf life of strawberries.

Khan et al.^19^ also concluded that strawberries coated with an edible antimicrobial chitosan coating showed less deterioration than those in the control group. The coated strawberries showed signs of decomposition after 6 days of storage; in the present study, such signs appeared in the control group on the seventh day.

Guerreiro et al.^39^ comment that the firmness of strawberries tends to decrease during storage due to cell wall degradation and loss of turgor. They also state that this is an acceptable quality parameter for fresh fruit. The researchers observed that the application of coatings based on polysaccharides, sodium alginate, and pectin, and compounds present in EOs, citral, and eugenol, improved the firmness of strawberries compared to the control group. However, they point out that the addition of more than 0.3% citral negatively affected the firmness of strawberries, and concluded that the concentration of certain compounds from EOs, which are included in the coating formulations, can lead to a reduction in fruit firmness.

The strawberries treated with EO-PB exhibited different characteristics to the other groups that did not receive EO treatment. The appearance of these strawberries was altered, with a less intense color compared to strawberries that did not receive the natural product (Figure 5).

**Figure 5:**
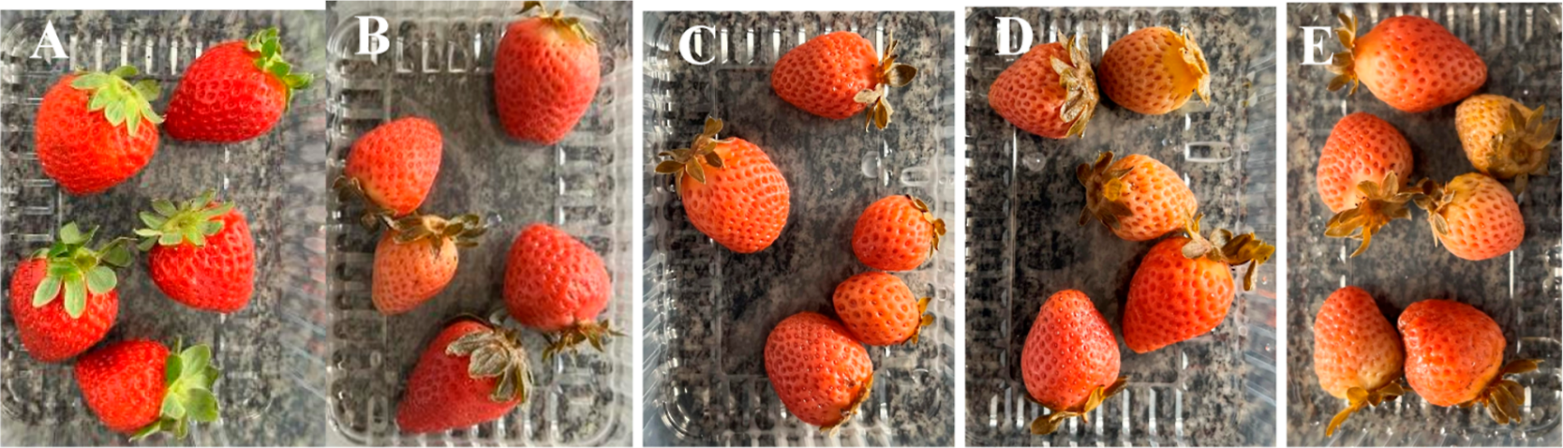
Group of strawberries on the day of the procedure (A), with 3 (B), 7 (C), 10 (D), and 15 (E) days after application of *Pectis brevipedunculata* essential oil.

The strawberry group treated with the ME-PB microemulsion exhibited color characteristics similar to those of the EO-PB group. However, these characteristics were not observed in the ME-WHITE group, suggesting that the color change was caused by the EO, which was also present in the ME, rather than by the ME surfactants.

Horison et al.,^40^ also observed a change in fruit color when evaluating strawberry coating with nutmeg seed oil nanoemulsion and chitosan; however, the observed change was from light red to dark red. They added that, even after incorporation into the fruit, the skin’s rigidity and color remained unchanged after 5 days of coating.

The pigments responsible for the color of strawberries, anthocyanins, have various structural forms, including flavilium cations, quinoid bases, carbinol pseudobases and chalcones, which exhibit different colors. The presence of certain factors, such as temperature, pH, light, oxygen, enzymes and possible bonds with other chemical substances, can affect these structures.^41,42^

Changes in these parameters during storage can cause oxidation or other types of chemical reactions involving anthocyanins. This can alter their synthesis or stability, resulting in the characteristic red color of strawberries being replaced by brownish, opaque pigments, or the fruit becoming discoloured.^42^

The pH level is an important factor in the stability and color expression of anthocyanins. Even small changes in pH can alter the chemical structure of anthocyanins, resulting in irreversible degradation and color fading.^43^

Holcroft and Kader^43^ observed that the pH increase caused by the high levels of CO_2_ gas used in the modified atmosphere resulted in the strawberries undergoing a fading or “whitening”.

Guerreiro et al.^39^ comment on the concentrations of components in antimicrobial coatings, as well as the damage that high concentrations of substances can cause to strawberry epidermal cells and the possible chemical reactions that can occur between the compounds in the formulation. Their studies revealed that these factors resulted in reduced firmness and increased weight loss in the fruit.

Taking the above into account, it can be inferred that the change in fruit color in the present study was caused by an increase in pH following the application of EO-PB, as can be seen in Table 4, and by the concentration of oil incorporated into ME-PB. While the theoretical contribution justifies the influence of pH on the expression of color by anthocyanins, the effect of the concentration of compounds on fruit coloration still needs to be proven. A prospect could be to incorporate a lower percentage of EO into the microemulsified system and observe color maintenance.

Even though the color appeared paler, to the detriment of the intense red typical of strawberries and the darker green tone of the leaves (see Figure 5), no apparent fungi were found during the experimental period. In other words, while the application of EO-PB and ME-PB did modify this visual characteristic, it did not affect shelf life, as the active ingredients contained in EO and ME prevented the proliferation of fungi.

The effectiveness of these bioproducts (EO-PB and ME-PB) is evident when their results are compared with those obtained from the application of sodium hypochlorite, a sanitizer frequently used in fruit sanitation. After 3 days, dark areas appeared on the fruit, signaling the development of microorganisms. After 10 days, fungi grew (Figure 4). Therefore, it can be inferred that EO-PB and ME-PB are more effective at inhibiting the growth of microorganisms than a 200 ppm sodium hypochlorite solution.

It is important to note that the storage recommendations of the manufacturer (Peterfrut, Espírito Santo, Brazil), as indicated on the strawberry packaging, were followed. The fruit should be stored at a temperature of 2 to 5 °C and washed before consumption. For the experiment, the strawberries were washed and sanitized before being packaged for evaluation. The manufacturer also notes that the strawberries should be consumed within 10 days.

It should be noted that a liquid was observed in the strawberry packaging in the formulation application group (ME-PB). It is inferred that this liquid is a microemulsion, given its similar color. It is suggested that, as this formulation is more viscous than the EO, it was not easily drained by the nylon filter during the material’s drying stage, resulting in excess sample on the fruit.

MEs can be applied to food by spraying, spreading, or immersion. To observe whether different results would be obtained by applying the sample differently, and to see how the excess sample in the packaging and the change in color of the strawberries would be affected, the spraying method was tested using a 100 mL container (Manancial do Brasil Importação e Comércio Ltd.a, Rio de Janeiro, Brazil) with a spray valve to spray the samples onto the fruit. After application, it was noted that the samples could be applied uniformly. However, over time, the strawberries changed color, becoming lighter, as with the immersion application. However, no excess sample was observed in the packaging (Figure 6). Based on these results, it is recommended that, in tests involving viscous formulations, the application method should be by spraying to prevent sample accumulation during the study.

**Figure 6:**
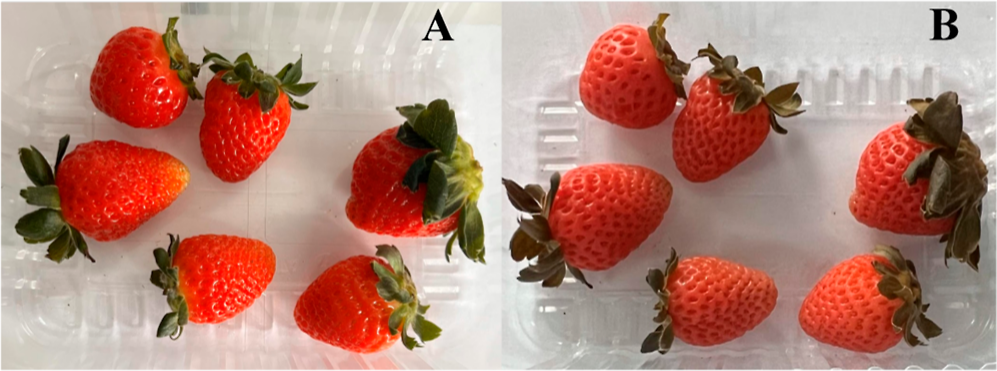
Group of strawberries on the day of the procedure (A) and 3 days (B) after spraying with *Pectis brevipedunculata* essential oil.

The quality of the strawberries in terms of bacteria and shelf life was verified by means of Total Mesophilic Aerobic Count on plates. Table 7 shows the mesophilic aerobes, in UFC/g, present in unsanitized strawberries, strawberries sanitized with a 200 ppm sodium hypochlorite solution, and strawberries treated with EO-PB, ME-PB, and ME-WHITE at all evaluation times. The investigation showed that the group of strawberries that was not sanitized had the highest bacterial count throughout the evaluation period. Proliferation increased from 7.1 × 10^2^ to 1.0 × 10^4^ UFC/g from the first to the last day of observation (see Figure 7; Table 7). Subsequently, the group of strawberries that were sanitized using a solution of a 200 ppm sodium hypochlorite solution presented the highest number of mesophilic aerobic colonies, increasing from 3.4 × 10^2^ to 8.6 × 10^3^ UFC/g. These results indicate that sanitization with sodium hypochlorite was insufficient to eliminate the microorganisms adhering to the fruit. An alternative antimicrobial product is therefore necessary, such as the natural product formulation proposed here.

**Table 7:** Total Count of Mesophilic Aerobes on Plates with Unsanitized Strawberries, Strawberries Sanitized with a 200 ppm Sodium Hypochlorite Solution, and Strawberries Treated with EO-PB, ME-PB, and ME-WHITE at all Evaluation Times^a^

	UFC/g				
time days	not sanitized	sanitized	EO-PB	ME-PB	ME-WHITE
0	7.1 × 10^2^	3.4 × 10^2^	0	0	1.2 × 10^2^
3	8.7 × 10^2^	4.4 × 10^2^	0	0	1.9 × 10^2^
7	2.2 × 10^3^	1.4 × 10^3^	0	0	3.5 × 10^2^
10	8.7 × 10^3^	1.8 × 10^3^	0	0	0
15	1.0 × 10^4^	8.6 × 10^3^	0	0	4.1 × 10^2^

a Source: Authors.

**Figure 7:**
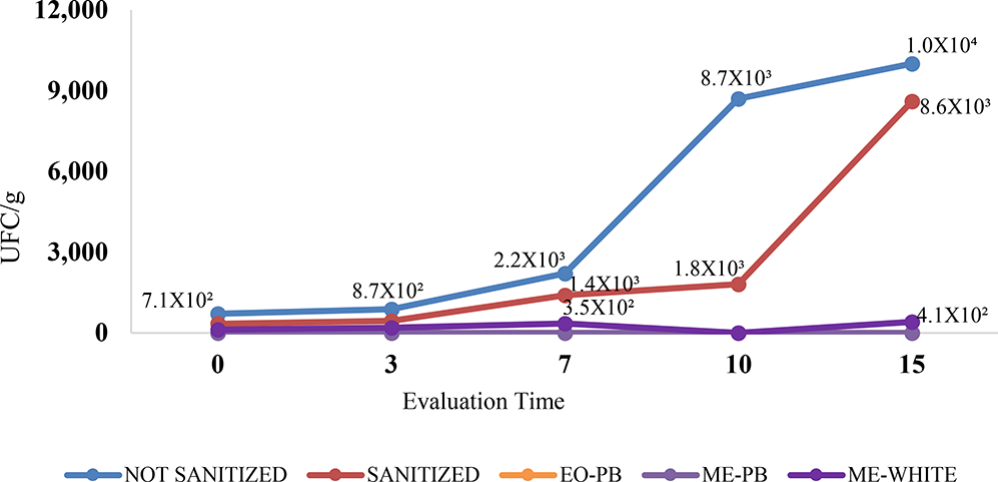
Graph showing the total count of mesophilic aerobes on plates with unsanitized strawberries, strawberries sanitized with a 200 ppm sodium hypochlorite solution, and strawberries treated with EO-PB, ME-PB, and ME-WHITE at all evaluation times.

No bacterial colonies were observed on the plates analyzed from any of the strawberry groups that received EO-PB and ME-PB applications at any time during the experiment. This means that EO-PB and ME-PB were able to inhibit the growth of microorganisms in the tested fruits. In contrast, the strawberries submerged in ME-WHITE developed bacteria almost constantly, proving that the antimicrobial action is due to the active ingredients in EO-PB that are present in ME-PB, rather than the surfactants in the formulation. The strawberries treated with ME-WHITE presented colonies of 1.2 × 10^2^ to 4.1 × 10^2^ UFC/g during the experiment (Figure 7; Table 7). There was no growth of mesophilic aerobes in the control of the standard agar count used.

Comparing the results achieved with those of other researchers, we found that Khan et al.,^19^ who researched chitosan-based antimicrobial coatings, also found that the number of microorganisms increased over the storage period, particularly in the control group, which ranged from 2.4 × 10^2^ to 2.1 × 10^5^ UFC/g (2.43 and 5.3 log UFC/g), these values being higher than those identified in the present study. The researchers concluded that using the antimicrobial coating they proposed resulted in fewer microorganisms developing, but there were still 1.1 × 10^2^ to 2.2 × 10^3^ UFC/g of mesophilic aerobes in the strawberries. Horison et al.^40^ also found that strawberries from the control group, stored at 10 °C for 5 days, had a quantity of mesophilic bacteria, 2.4 × 10^3^ UFC/g (3.37 ± 0.02 log UFC/g), higher than strawberries immersed in a nanoemulsion of nutmeg seed oil, chitosan, and Tween 80, which was 2.7 × 10^2^ UFC/g (2.41 ± 0.01 log UFC/g).

Guerreiro et al.^39^ identified aerobic mesophilic counts in the control group of strawberries ranging from 8.4 × 10^2^ to 4.2 × 10^5^ UFC/g (2.9 to 5.6 log 10 UFC/g) colonies. In the group of strawberries coated with the formulation of 2% sodium alginate + 0.15% citral + 0.10% eugenol, the count decreased from 8.4 × 10^2^ UFC/g (2.9 log 10 UFC/g to zero) to zero mesophilic aerobic colonies. In the present study, no growth of microorganisms was observed in the strawberry groups treated with EO-PB or ME-PB at any time during the experiment. Therefore, our results are promising compared to those reported in the literature.

Horison et al.^40^ observe that the antimicrobial action of EO can be optimized through nanoscale encapsulation, as this results in a higher concentration of bioactive principles in the region where microorganisms are present.

In the present study, the situation observed 10 days after application of ME-WHITE, in which there was a significant reduction in the growth of microorganisms, followed by a resurgence in growth 15 days later, was also observed by Guerreiro et al.^39^ at 7 and 14 days into the experiment with strawberries coated with sodium alginate and pectin-based formulations.

The presence of fungal infection was also analyzed in the strawberry groups (Table 8; Figures 8 and 9). Using the mold and yeast enumeration technique and the depth plating method, it was found that the most intense fungal proliferation occurred in strawberries that had not been sanitized. Table 8 presents fungal proliferation in unsanitized strawberries, strawberries sanitized with 200 ppm sodium hypochlorite solution and strawberries treated with EO-PB, ME-PB and ME-WHITE at all evaluation times. In unsanitized strawberries, the highest number of fungal colonies was observed throughout the evaluation period, starting at 2.7 × 10^2^ UFC/g at time 0 and rising to 6.9 × 10^3^ UFC/g on the 15th day of the experiment. The group of strawberries sanitized with sodium hypochlorite solution also stood out for presenting fungi at practically all evaluation times. In the first analysis period, this group presented 1.5 × 10^2^ UFC/g, which was reduced to zero in 3 days. After 7 days, the value rose to 1.6 × 10^3^ UFC/g, and after 10 days, there was a further reduction in the number of fungi to 3.6 × 10^2^ UFC/g and in the last analysis period, the value increased, reaching 7.3 × 10^2^ UFC/g. The strawberries treated with ME-WHITE developed fungi after 3 days (1.4 × 10^2^ UFC/g), 7 days (1.6 × 10^2^ UFC/g), and 15 days (2.3 × 10^3^ UFC/g) (Table 8; Figures 8 and 9). The strawberries that were submerged in the application of EO-PB and ME-PB did not show fungal infection, indicating the action of both EO and the formulation against molds and yeasts. Similarly, there was no fungal growth in the Sabouraud Dextrose Agar control.

**Table 8:** Fungal Proliferation in Unsanitized Strawberries, Strawberries Sanitized with a 200 ppm Sodium Hypochlorite Solution, and Strawberries Treated with EO-PB, ME-PB, and ME-WHITE at all Evaluation Times^a^

	UFC/g				
time days	not sanitized	sanitized	EO-PB	ME-PB	ME-WHITE
0	2.7 × 10^2^	1.5 × 102	0	0	0
3	6.4 × 10^2^	0	0	0	1.4 × 10^2^
7	2.3 × 10^3^	1.6 × 10^3^	0	0	1.6 × 10^2^
10	2.7 × 10^3^	3.6 × 10^2^	0	0	0
15	6.9 × 10^3^	7.3 × 10^2^	0	0	2.3 × 10^3^

a Source: Authors.

**Figure 8:**
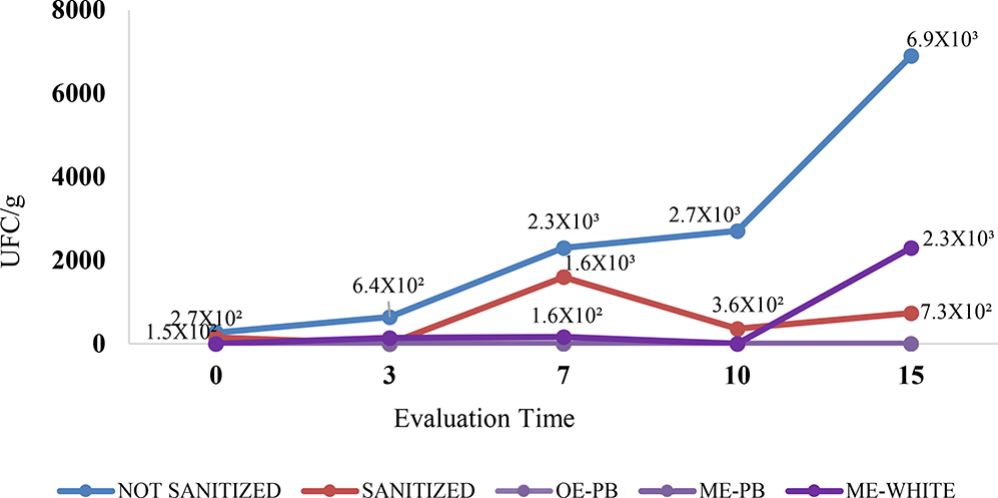
Graph showing fungal proliferation in unsanitized strawberries, strawberries sanitized with a 200 ppm sodium hypochlorite solution, and strawberries treated with EO-PB, ME-PB, and ME-WHITE at all evaluation times.

**Figure 9:**
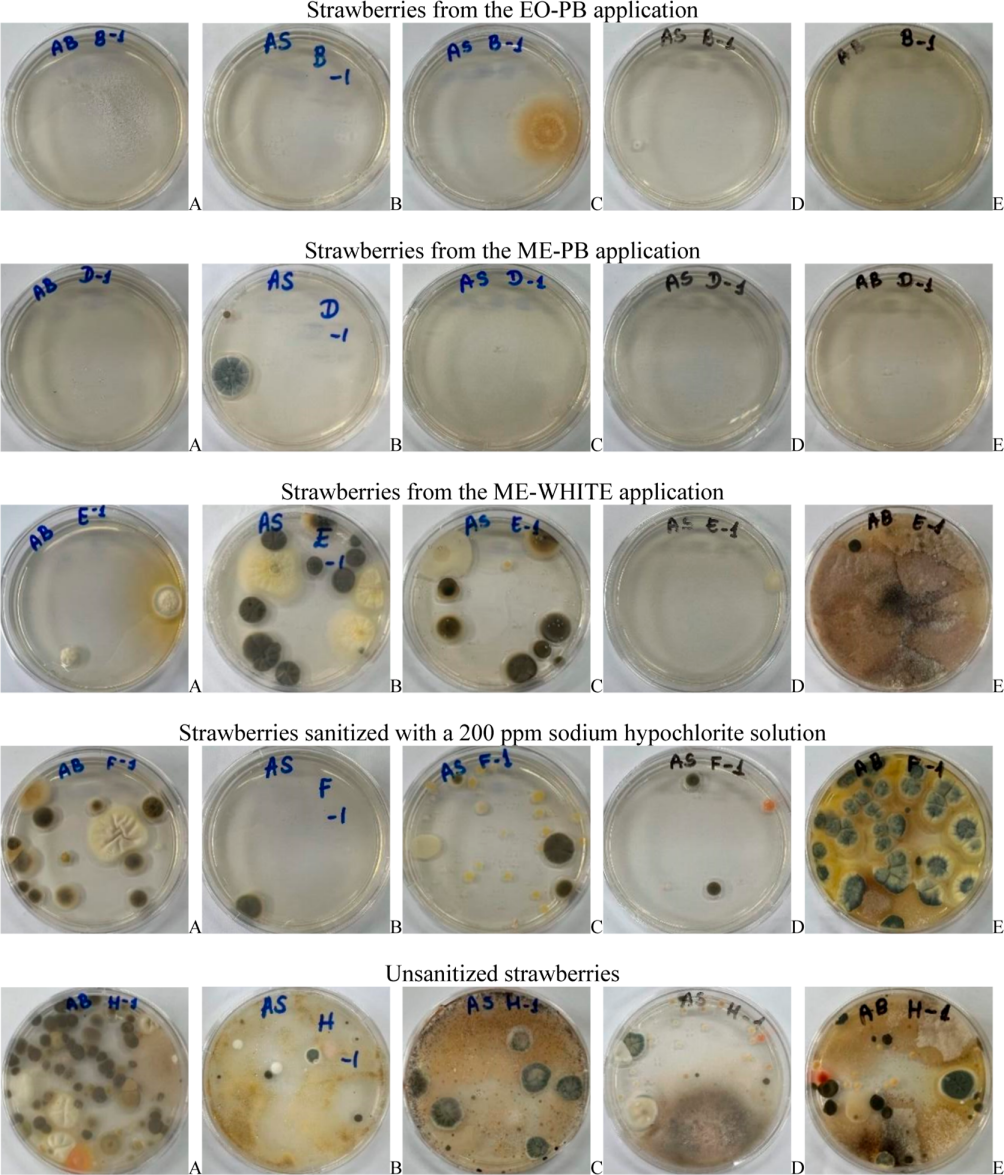
Evaluation of fungal proliferation from strawberries after application of EO-PB, application of ME-PB, application of ME-WHITE, strawberries sanitized with a 200 ppm sodium hypochlorite solution and unsanitized strawberries, at time zero (A), 3 (B), 7 (C), 10 (D) and 15 (E) days after the procedure.

EO and the formulation inhibited the proliferation of molds and yeasts in strawberries. While the formulation was as efficient as the essential oil (EO) at inhibiting growth, using the microemulsified system (ME-PB) is advantageous as it allows a smaller volume of EO to be used to achieve the same results as using EO in its pure form, which requires a larger volume. This is an interesting strategy considering that the yield of EO extracted from the aerial parts of *P. brevipedunculata* is only around 0.67%.

Microemulsion systems are also advantageous because they facilitate the solubilization of compounds, particularly lipophilic ones such as essential oils.^17^ Microemulsions (MEs) are an alternative system that can increase the chemical stability of secondary metabolites present in essential oils (EOs), decrease losses of compounds through volatilization and reduce the toxicity of the compounds.^17,44^

In addition to these applications, microemulsions (MEs) optimize antimicrobial potentials because they increase product diffusion, generating greater contact with microorganisms and potentially interfering with biofilm formation.^45,46^ This antimicrobial action extends to fungi, as observed by Basak and Guha^47^ in the ME based on Betel essential oil (*Piper betle* L.), which showed an inhibitory effect on the growth of *Aspergillus flavus* in tomatoes. Based on the results, it can be inferred that ME can be considered a potential vehicle for the release of bioactive principles in food.

Strawberries are predisposed to contamination by molds and yeasts. Factors such as high levels of sugars and other nutrients; water activity ideal for microbial growth; low pH; and a fragile surface layer that can be easily damaged, allow for the emergence and increasing development of molds and yeasts.^18,19^

In order to compare the results obtained in this work with the literature, we checked that Khan et al.^19^ also noted an increase in the number of mold and yeast colonies in strawberries over time, ranging from 2.1 × 10^2^ to 3.3 × 10^6^ UFC/g (2.34 and 6.49 log UFC/g). The researchers initially recorded lower values than those we noted, but at the end of the test they reached values higher than those found in the present study, since in the last evaluation period we obtained the equivalent of 6.9 × 10^3^ UFC/g for unsanitized strawberries. And in strawberries with antimicrobial coating, Khan et al.^19^ enumerated 1.6 × 10^2^ to 1.1 × 10^5^ UFC/g (2.22 and 5.04 log UFC/g). Horison et al.^40^ found fungi in the order of 6.3 × 10^2^ UFC/g (2.78 log UFC/g) in coated strawberries stored at 10 °C for 5 days. Guerreiro et al.^39^ observed, over a 14 day period, in strawberries coated with the formulation of Pectin 2% + Citral 0.15% + Eugenol 0.10%, a reduction in fungi from 3.0 × 10^2^ to 2.1 × 10^1^ UFC/g (2.5 to 1.3 log 10 UFC/g). Meanwhile, in strawberries treated with EO-PB and ME-PB, there was no fungal growth or proliferation. This demonstrates the potential of the tested EO and ME.

Guerreiro et al.^39^ also observed the same situation in one of their coatings as occurred at time point 10 in the present study after the application of ME-WHITE, where there was a significant reduction in microbial growth. The next time they checked, 15 days later, growth had increased again.

According to the results, mesophilic aerobes were present in all treatment groups at the start of the experiment (1 h after the samples were applied), except in the EO-PB and ME-PB immersion groups (see Table 7). Regarding fungi, they were also absent from the EO-PB, ME-PB and ME-WHITE groups at the initial time point. These findings demonstrate that the essential oil and formulation samples were both effective in eliminating microorganisms within 1 h, which was considered the zero time period. A similar situation was observed by Khan et al.,^19^ who demonstrated that applying the antimicrobial coating at the zero time point minimized the levels of mesophilic aerobes, molds and yeasts.

The presence of microorganisms such as molds, yeasts and mesophilic bacteria in fresh fruit such as strawberries has been reported in the literature. Colonies of 10^3^ to 10^9^ UFC/g of these microorganisms can originate in the field at harvest time, or during transport, marketing, or even during handling by the consumer. Thus, there is a possibility that fresh fruits may reach the consumer already carrying a microbial load, which must be eliminated through sanitization procedures.^48,49^ It was noted that the amount of fungi present at the initial time of the experiment was eliminated by sanitization, which prevented new proliferation after 3 days. However, after 7 days, sodium hypochlorite failed to contain the development of the microorganism. According to Rodrigues et al.,^50^ the sanitizer can only reduce the microbial load by up to two logarithmic cycles. This reduction in competitive microbiota may allow the development of pathogens.

In the present study, the following fungal genera were identified in strawberries: *Curvularia*, *Cladosporium*, *Aspergillus*, *Penicillium*, *Purpureocillium*, and *Talaromyces* (Figure 2S). Fungal identification occurred after 7 days of growth, where macroscopic characteristics were observed (Table 1S): diameter, texture, pigmentation, surface, edge, topography, color, aspect, pigment, and growth; and microscopic characteristics of morphological structures (Table 2S): hypha, conidium, conidiophore, phialide, foot cell, and vesicle. The identification of filamentous fungi at the genus level followed the recommendations of the book “Larone’s medically important fungi: a guide to identification”.^51^

Of the fungi detected, some are defined in literature as the most common found in strawberries, such as fungi of the genera *Penicillium* and *Cladosporium*. These fungi cause diseases in the fruit, associated with postharvest lesions.^49^ Souza^52^ cites the species *Penicillium expansum*, *Penicillium purpurogenum* and *Aspergillus niger* as the main phytopathogenic agents causing advanced deterioration in strawberries. Fungi belonging to the genus *Talaromyces* deserve some attention, as they are able to produce heat-resistant spores, and for this reason they can survive the heat treatments of food processing.^53^

Tournas, Heeres and Burguess^54^ also identified the presence of *Cladosporium* in strawberry salads. Tournas and Katsoudas^49^ verified that fungal species of *Cladosporium* created dark spots and *Penicillium* fungi produced a soft rot in strawberries, which were covered with green or bluish conidia and had a strong, unpleasant odor. Souza^52^ identified the fungal species *Purpureocillium lilacinus* in strawberry cultivation systems. The fungus was obtained from *Lobiopa insularis*, one of the main pests of strawberry crops, and similarly, fungi of the genera *Talaromyces* (*T. amestolkiae* and *T. verruculosus*), *Aspergillus* (*Aspergillus nomius* and *Aspergillus westerdijkiae*), and *Penicillium* (*Penicillium brasilianum*, *Penicillium citrinum*, *Penicillium commune*, *Penicillium janthinellum* and *Penicillium paxilli*) were identified. These genera were recorded in the present work.

The literature does not mention the *Curvularia* genus in relation to strawberries. Many species of this genus of fungi are pathogenic and cause a variety of plant diseases, frequently resulting in leaf spots and losses in agricultural production.^55^ The morphological characteristics identified in the present study are in accordance with those cited by Santos et al.^55^

Some of the fungi mentioned can produce mycotoxins, which can cause health risks such as allergic reactions.^49^ Reports in the literature suggest that *Penicillium* and *Aspergillus* species of fungi can grow and produce mycotoxins at room temperature, and even at low temperatures.^49^ Based on this data, it can be concluded that rigorous antifungal control is essential for strawberries and that new measures must be sought and implemented, such as the use of natural products.

The literature contains studies reporting on emulsion techniques and edible coatings based on polysaccharides, essential oils, and plant extracts as sources of bioactive compounds, incorporated with antimicrobial and antioxidant agents, which optimize the preservation of foods such as strawberries. Studies demonstrate the enhanced properties and confirm the effectiveness of these coatings with nano- and microencapsulation of bioactives, configuring themselves as advanced strategies to increase the shelf life of food. Table 3S presents different types of formulations, with various incorporated compounds applied to strawberries, to investigate diverse biological and chemical actions.

The EO-PB, as well as the ME-PB formulation, has citral among its major compounds. Citral exhibits biological activity, such as fungicidal activity. Marques et al.^6^ tested the action of *P. brevipedunculata* EO against fungi (*A. niger*, *Candida albicans*, *Cryptococcus neoformans*, *Fonsecaea pedrosoi*, *Microsporum canis*, *Microsporum gypseum* and *Trichophyton rubrum*), confirming the inhibitory activity of *P. brevipedunculata* EO. Corrêa et al.^56^ evaluated the effect of *P. brevipedunculata* EO in controlling the fungus *Colletotrichum gloeosporioides* in mangoes, concluding that the EO is efficient in controlling fungal diseases in mangoes.

Citral develops antifungal mechanisms capable of altering hyphal morphology and mycelial distortion, increasing fungal membrane permeability, causing the release of cellular constituents, leakage of potassium ions, inducing a decrease in the total lipid and ergosterol content of cells, indicating a disruption of membrane integrity.^57^

The compound Limonene, classified among the major compounds of EO-PB, also has proven fungicidal activity, being efficient in inhibiting *Aspergillus* species, and can completely inhibit the growth of *A. flavus*, an aflatoxin-producing fungus, at a concentration of 500 ppm.^18^

Based on the presented results, it can be inferred that EO-PB and ME-PB exhibited promising antifungal activity, thereby extending the shelf life of strawberries during storage. Further studies are needed to optimize the application of the developed essential oil (EO) and methanolic extract (ME-PB) in order to use them as a coating material in the food industry.

Based on the results obtained with the microemulsion, it can be inferred that it has the potential to be used as a regulated preservative in the food industry. However, for microemulsions to be used in food products, the surfactants in their composition must be approved by relevant bodies such as the U.S. Food and Drug Administration (FDA), the European Food Safety Authority (EFSA) and the Brazilian National Health Surveillance Agency (ANVISA).

Transcutol P is listed on the FDA’s List of Food Additives^58^ and in ANVISA Resolution RDC N° 725, of July 1, 2022, which deals with flavoring food additives. It functions as a flavoring diluent to facilitate their incorporation and dispersion in foods, and can be used to encapsulate flavorings in order to protect them from evaporation and possible alterations.^59^

Polysorbates are listed in the List of Food Additives Authorized for Use in Food in Brazilian Legislation, IN N° 211, of March 1, 2023, from ANVISA.^60^ The maximum permitted limits of polysorbates vary from 1000 to 10,000 mg/kg and the technological function alternates between emulsifier and stabilizer, depending on the food.^60^ Polysorbates are also approved as additives by the Food and Agriculture Organization of the United Nations (FAO), with a recommended Acceptable Daily Intake (ADI) of 25 mg/kg of body weight/day. This recommendation stems from the low acute toxicity of these compounds, with genotoxicity, carcinogenicity, or developmental toxicity not being a concern.^61^ Polysorbates are one of the most widely used hydrophilic surfactants in the food industry, along with proteins and carbohydrates with high molecular weight.^15^

Based on the above, it can be inferred that the use of MEs with the incorporation of active substances to protect food constitutes a strategy to guarantee the food’s resistance against contaminating and spoilage agents, extend the food’s shelf life, and consequently, reduce costs associated with food preservation, ensure consumer acceptance, and maximize profits for producers and retailers.

## 3 Conclusions

The incorporation of *P. brevipedunculata* essential oil into a microemulsion markedly enhanced the bioavailability and antimicrobial efficacy of its major constituents (α-pinene, limonene, neral, and geranial). The resulting formulation exhibited stronger antimicrobial activity than the free essential oil, with lower inhibitory concentrations against key foodborne pathogens, and effectively suppressed microbial and fungal growth on strawberries, extending their shelf life. These findings demonstrate that microemulsification is an efficient strategy to potentiate the biological activity and stability of essential oils, highlighting the biotechnological potential of this system for food preservation applications.

## 4 Materials and Methods

### 4.1 Collection of Plant Material

The plant material (aerial parts) of *P. brevipedunculata* was collected at the São Luís-Bacanga Campus of UFMA, São Luís, MA, Brazil (coordinates: 2°55′21.6″s; 44°30′71.0″w). The botanical identification of the species is cataloged in the Rosa Mochel Herbarium, São Luís, Maranhão, Brazil, registered under number 5287; and in the Maranhão Herbarium, with registration number 11,090. This species is registered in the National System for the Management of Genetic Heritage and Associated Traditional Knowledge (SISGEN) under registration number AAFB38B.

### 4.2 Obtaining the Essential Oil

The essential oil (EO) of *P. brevipedunculata* (EO-PB) was extracted by hydrodistillation using the Clevenger system.^20,62,63^ Initially, to obtain the oil, the plant sample underwent a drying process at room temperature for 48 h. Then, the sample was fragmented using scissors, 300 g of the sample was added to a 6000 mL round-bottom flask, submerged in distilled water and subjected to a temperature of 100 °C for 2.5 h. After this period, the essential oil (EO) was centrifuged (Centrifuge Centribio, model 80-2B, São Paulo, Brazil) at 3500 rpm (revolutions per minute) for 10 min to separate the oily and aqueous phases, and anhydrous sodium sulfate P.A. (Isofar, Rio de Janeiro, Brazil) was used to dry the residual water. The EO was stored in a glass vial, protected from light and in a refrigerated environment.

The yield of the EO was calculated based on the dry weight of the plant material using eq 1 ^64^

1 $$yield=\frac{V_{OE}\times \rho_{OE}}{m_{sample}-\left(m_{sample}\times \%\;moisture\right)}\times 100$$

where, *V*_EO_ = volume of extracted essential oil; ρ_EO_ = density of essential oil; *m*_amostra_ = mass of the dry plant sample; % moisture = moisture content of the dry plant sample.

Given that the density of the EO is calculated using eq 2 based on the mass (*m*_EO_) and volume (*V*_EO_) of the EO

2 $$\rho_{OE}=\frac{m_{OE}}{V_{OE}}$$

The moisture content of the plant sample was determined before the EO extraction, and this was verified using an Infrared Moisture Analyzer (GEHAKA, model IV2500, São Paulo, Brazil). The parameters used were: sample mass: 2 g; analysis temperature: 115 °C; and drying rate of 0.01%/ minute.^65^

### 4.3 Development of the Microemulsion and Incorporation of the Essential Oil

The development of the microemulsion was carried out according to Lopes et al.^20^ The surfactants Tween 80 (Polysorbate 80, Êxodo Cientifica, São Paulo, Brazil) and Transcutol P (Diethylene glycol monoethyl ether, Êxodo Cientifica, São Paulo, Brazil) were used. The ME was formulated by mixing the surfactants in a 1:1 ratio under magnetic stirring (SolidSteel, Piracicaba, Brazil) for 2 min at room temperature, as recommended by Do Nascimento et al.^26^ Subsequently, the oil phase, the EO, was titrated with the surfactants in the proportions (weight/weight) 1:9; 2:8; 3:7; 4:6; 5:5; 6:4; 7:3; 8:2 and 9:1, under magnetic stirring for 3 min. For each proportion, distilled water was slowly titrated in quantities of 1, 2, 3, 4, 5, 6, 7, 8, and 9, with constant magnetic stirring for 10 min. The phase behavior of the pseudoternary system of the 81 formulations was monitored using a black background for virtual inspection, as described by Silva et al.^66^ The stability of the systems was verified after 24 h of rest at room temperature. Next, based on the recorded changes in visual appearance, a pseudoternary phase diagram was constructed, plotting the points in Origin Pro 8.6 software (OriginLab Corporation, Northampton, USA). The diagram highlights the Winsor Classification regions: WI (Winsor I, two-phase system formed by an oily phase in equilibrium with an emulsified phase); WII (Winsor II, two-phase system formed by an aqueous phase in equilibrium with an emulsified phase); WIII (Winsor III, three-phase system formed by an aqueous phase and an oily phase, interspersed by an emulsified phase); WIV (Winsor IV, single-phase system, the microemulsion region);^22^ in addition to milky white liquid emulsion and turbid system. Based on the analysis of the phase diagram, the ideal ME formulation was defined. The formulated ME was stored in a place protected from light, heat and humidity, and after 48 h, according to the indication of Silva et al.,^66^ it was chemically and biologically characterized.

### 4.4 Chemical Characterization of Essential Oil and Microemulsion

The analysis of the EO was performed using a Gas Chromatograph (GC-2010) coupled to a Mass Spectrometer (GC–MS) (GCMS-QP2010 Plus) (Shimadzu, Kyoto, Japan). The chromatographic conditions used were as described by Lopes et al.^20^ The sample was prepared with 2 μL of EO diluted in 500 μL of *n*-hexane. A DB-5MS capillary column (30 m × 0.25 mm × 0.25 μm) was used with helium as the carrier gas, at a linear velocity of 39.5 cm/s and a column flow rate of 1.0 mL/min, split ratio 1/30. The furnace program was set to 40 °C with a heating ramp of 10 °C/min up to 280 °C. The injector and ion source temperatures were 250 and 200 °C, respectively. Retention indices were calculated for all volatile components using a homologous series of C8–C20 *n*-alkanes (Sigma-Aldrich, St. Louis, Missouri, USA), according to the Van Den Dool and Kratz linear equation.^67^ The EO components were identified by comparing their retention indices and mass spectra (molecular mass and fragmentation pattern) with data stored in the Adams^68^ and NIST (National Institute of Standards and Technology)^69^ libraries.

The EO and ME were analyzed by GC/MS-headspace, according to Lopes et al.,^20^ using the following chromatographic conditions for volatiles: cycle: HS-INJ; sample volume: 50 μL of EO and 500 μL of ME; syringe: 2.5 mL—HS; incubation temperature: 85 °C; incubation time: 5 min; agitation speed: 250 rpm; agi on time: 10 s; agi off time: 2 s; syringe temperature: 85 °C; filling speed: 100 μL/s.

The EO and ME were further characterized by Fourier Transform Infrared (FTIR) Absorption Spectroscopy, where absorption spectra in the mid-infrared region were obtained using an IR Tracer 100 instrument (Shimadzu, Quito, Japan), in the range of 4000 to 600 cm^–1^, with a resolution of 4 cm^–1^ and an accumulation of 50 spectra per verification. The sample window used was ZnSe and the sample volume was 20 μL. The interpretation of the spectra was performed according to Donald et al.^21^ and Lopes and Fascio.^70^

### 4.5 Physicochemical Characterization of the Microemulsion

The physicochemical characterization of the microemulsion was performed by Lopes et al.,^20^ in which the researchers verified the appearance of the microemulsion regarding transparency, absence of phase separation, and the color of the formed system. Lopes et al.^20^ analyzed the droplet size (average diameter z), the polydispersity index (PDI), and the zeta potential using a Zetasizer Nano ZS90 (Malvern Instruments, Malvern, United Kingdom), performed a thermal stability test of the ME, and verified the density, pH, and refractive index, and the maintenance of these physical parameters after thermal variation.

To complement the physicochemical characterization of the formulation, in the present study, the freeze–thaw test was performed again,^16^ however, the objective of this step was to investigate the thermal stability of the ME in relation to its chemical composition and its biological activity. Thus, the test was performed before and after the infrared analysis of the ME and microbiological analysis, in order to confirm that the active principles present in the formulation composition and the antimicrobial activity^71–73^ remained with the temperature variation.

The physical stability of the ME, as well as its consistency and the mechanical properties of the fluid, were determined by rheological analysis. Rheology was performed according to Do Nascimento et al.^26^ A controlled stress rheometer (MARS II, Haake Thermo Fisher Scientific Inc., Germany) was used, with a cone/steel plate geometry (C35/2° Ti) of 35 mm in diameter, with a fixed distance of 0.105 mm between the plates. The sample evaluation temperature was 25 °C. The sample resting time was 1 min. Shear rates were varied from 0 to 500 s^–1^ and from 500 to 0 s^–1^ to obtain the flow curves. The curves were measured every 150 s.

### 4.6 Evaluation of the Antimicrobial Activity of Essential Oil and Microemulsion

The antimicrobial activity was performed using microbial strains from the American Type Culture Collection (ATCC), donated by the Microbiology Laboratory of Food and Water Quality Control at UFMA (PCQA-UFMA): *E. coli* (ATCC 25922), *Salmonella* spp. (ATCC 14028), *L. monocytogenes* (ATCC 35152), *S. aureus* (ATCC 25923), *B. cereus* (ATCC 14579) and *Clostridium* spp. (ATCC 25772).

For inoculum standardization, the microbial strains used were subcultured in tryptone soy broth (TSB) (Merck, Darmstadt, Germany) and incubated in a bacteriological culture incubator (Fanem, São Paulo, Brazil) at 35 °C until they reached the exponential growth phase (4 to 6 h). After this period, the cell density of the cultures was adjusted in sterile 0.85% saline solution, in order to obtain a microbial suspension with turbidity comparable to that of McFarland standard solution 0.5 (1.5 × 10^8^ UFC/mL) according to the Clinical and Laboratory Standards Institute standards.

The antimicrobial activity of EO and ME was determined by the disk diffusion method (DDM)^71,73,74^ and by broth dilution, resulting in the minimum inhibitory concentration (MIC).^72,73^

The experiments were conducted using a biological replica. Regarding the nature of the technique’s replication, each sample was analyzed in triplicate. Differences between means were determined using Tukey’s test, with a significance level of *p* ≤ 0.05.

In the disk diffusion test, triplicate Petri dishes were used for each bacterium to be tested; that is, Mueller Hinton agar (Merck, Darmstadt, Germany) was added to each Petri dish, and the bacterial suspensions were distributed. In this way, the samples are considered independent, as they are cultivated under similar conditions, however, they are treated separately, which guarantees consistent results.

In the bacterial inhibition assay using DDM, Mueller Hinton Agar (Merck, Darmstadt, Germany) was used to distribute 75 μL of the microbial suspension. Antibiotic susceptibility testing discs (Laborclin, Paraná, Brazil), 6 mm in diameter, containing 50 μL of the EO, ME and ME-WHITE (Microemulsion blank, i.e., formulation developed with surfactants and distilled water, without the addition of essential oil) samples were placed on the surface of the culture medium. The samples were added to the discs undiluted. The EO is incorporated into the microbiota at a concentration of 200 mg/mL. The positive control was performed with the commercial antibiotics Gentamicin (Laborclin, Paraná, Brazil) and Penicillin (Laborclin, Paraná, Brazil). A negative control was also performed, in which the microbial suspension was distributed on the surface of Mueller Hinton agar (Merck, Darmstadt, Germany) and bacterial growth was observed. A control with ME-WHITE was also performed, where the antimicrobial action of the formulation components was verified. The plates were incubated in a bacteriological incubator at 35 °C for 24 h. After this period, the diameters of the inhibition halos were measured. This assay was performed in triplicate.^71,74^

The bacterial inhibition verified by the MIC was determined according to the broth dilution methodology, proposed by the National Committee for Clinical Laboratory Standards.^72^ A solution was prepared containing the sample (EO, ME and ME-WHITE) and dimethyl sulfoxide (DMSO) (Êxodo cientifica, São Paulo, Brazil) at 0.1%, considering the concentration of 200 mg/mL of the EO in the ME. An aliquot of 1000 μg/mL of the EO, ME and ME-WHITE solutions was added to Mueller Hinton broth (Merck, Darmstadt, Germany).

Serial dilutions were then performed, resulting in concentrations of 12,000 to 15,625 μg/mL (12,000; 10,500; 10,000; 8000; 6000; 5000; 4000; 3500; 3000; 2500; 2000; 1500; 1000; 500; 250; 125; 62.5; 31.25; 15.63 μg/mL). A microbial suspension containing 1.5 × 10^8^ UFC/mL of bacteria was added to each concentration. A negative control was performed to verify bacterial growth, and a positive control was performed with the commercial antibiotics ampicillin and gentamicin, at concentrations of 128 to 0.015625 μg/mL (128; 64; 32; 16; 8; 4; 2; 1; 0.5; 0.25; 0.125; 0.0625; 0.03125; 0.015625 μg/mL). DMSO and broth controls were also performed. The tubes were incubated at 35 °C for 24 h.^75^ After the incubation period, the MIC of the EO and ME was verified, which was detected by the absence of visible turbidity in the tubes.

The MIC was also verified by the colorimetric method using the resazurin marker (7-hydroxy-3*H*-phenoxazine-3-one-10-oxide) (Merck, Darmstadt, Germany) to indicate the presence of viable cells, according to Chada,^76^ with adaptations. After the incubation period, 20 μL of sterile aqueous solution of 1% resazurin was applied at the tested concentrations, incubated in an oven at 35 °C for 30 min, and then the color change was observed. Resazurin has a blue color, and in the presence of viable cells the compound undergoes reduction, transforming into resofurin and the color changes to pink (Figure 1S).

Therefore, the concentrations that exhibited a pink coloration indicate microbial growth, due to the inactivity of the EO and ME under study, demonstrating that they do not possess bactericidal action. The concentrations that showed a blue coloration demonstrated inhibition of microbial growth. Thus, the MIC was the lowest concentration that did not change the total or partial color of the medium, remaining blue.^76^

### 4.7 Application of Essential Oil and Microemulsion to Strawberries

The application of EO and ME in food was carried out according to the recommendations of Khan et al.^19^ and Arnon-Rips et al.^77^ Strawberries (*Fragaria vesca* L.) (Peterfrut, Espírito Santo, Brazil), acquired from the Maranhão Supply Centers SA (Ceasa-MA), were previously selected for the test according to uniformity of size, weight, color and absence of physical or pathological damage.

The fruits were washed with running water, immersed in a sodium hypochlorite solution (Start, Rio de Janeiro, Brazil) at 200 ppm for 15 min for decontamination, and then rinsed with potable water and dried at room temperature on a nylon filter (Sbrissa, São Paulo, Brazil). Groups of 5 (five) strawberries, in triplicate, were immersed in the samples (EO, ME and ME-WHITE) for 30 s, to create a thin coating on the surface of the fruit, then dried for 1 h at room temperature, after which the strawberries were placed on a nylon filter to drain the excess samples.

Unsanitized strawberries and strawberries sanitized in a sodium hypochlorite solution constituted the control groups. The fruits were packaged in polyethylene terephthalate (PET)/Low-density polyethylene (LDPE)/Polypropylene (PP) containers (PRAFESTA IND. E COM. DE EMBALAGENS LTDA. São Paulo, Brazil) and stored at 5 °C in a refrigerator (Electrolux, São Paulo, Brazil). The strawberries were examined on the day of processing (time 0) and at four other times, at 3, 7, 10, and 15 days, in relation to visual decay, rate of spoilage, weight loss, moisture, total soluble solids content, and pH.

Visual decay was examined in strawberries, and those showing visible, dark-colored lesions, characteristic of an area infected by microorganisms, were considered deteriorated, according to Khan et al.^19^ The strawberry rot rate was calculated by dividing the number of rotten fruits by the initial number of fruits and multiplying by 100.^78^ Strawberry weight loss was expressed according to Khan et al.,^19^ who consider the percentage loss based on the initial and final weight. Strawberry weight loss was calculated using eq 3

3 $$weight\;loss\;\left(\%\right)=\left(A-\frac{B}{A}\right)\times 100$$

where *A* is the initial weight and *B* is the final weight of the strawberry.

Moisture content was determined by direct drying in an oven (SolidSteel, Piracicaba, Brazil) at 105 °C until dry weight was obtained.^79^ Total soluble solids content was determined using an Abbe benchtop refractometer (QUIMIS, model Q767B, São Paulo, Brazil) at 20 °C, and the results were expressed as °Brix.^79^ The pH of the strawberries was evaluated using a digital pH meter (BEL engineering, model PHS3BW, Piracicaba, Brazil) according to the standard method described in the Adolfo Lutz Institute.^79^

For the microbiological characterization of the strawberries, 10 ± 0.2 g of strawberries from each treatment were weighed and added to 90 mL of sterile 0.1% saline solution, prepared with sodium chloride (Ciavicco, Arraial Velho, Brazil), corresponding to the first dilution (10^–1^). Subsequently, successive dilutions of 10^–2^ and 10^–3^ were prepared in test tubes containing 9 mL of 0.1% saline solution.

The quality of strawberries in relation to bacteria and shelf life was investigated by Total Aerobic Mesophilic Plate Count, also known as Standard Plate Count. The method used was pour plating, where samples were inoculated onto sterile Petri dishes by pouring approximately 15 mL of standard plate count agar (PCA) (Merck, Darmstadt, Germany), mixing the inoculum with the culture medium by gently moving the plates, and then allowing the medium to cool and solidify. Agar control was performed. Plates were incubated in a bacteriological incubator (Fanem, São Paulo, Brazil) at 37 °C for 24 h. Afterward, microbial growth was evaluated and colony counts were performed using a colony counter for Petri dishes (Phoenix, São Paulo, Brazil). Results were expressed in Colony Forming Units (UFC) per gram. The procedures followed the recommendations of the American Public Health Association.^80^

Strawberries were also investigated for fungal infection. The mold and yeast enumeration technique and the pour plate method were used, where 0.1 mL of serial dilutions (10^–1^, 10^–2^, and 10^–3^) and 15 mL of Sabouraud Dextrose Agar (KASVI, Paraná, Brazil) acidified with 10% tartaric acid solution were added. The agar was controlled. After the procedure, the plates were incubated in a BOD incubator—Biochemical Oxygen Demand (HydroSan, São Paulo, Brazil) at 25 °C ± 2 °C for 5 days. The results were expressed in UFC/g. The analyses followed the recommendations of the American Public Health Association.^81^

Macroscopic identification of filamentous fungi occurred after 7 days of growth, where macroscopic characteristics such as surface, texture, topography, pigmentation, among others, were observed and classified by the presence or absence of filamentous fungi in the fungus, according to the book “Larone’s medically important fungi: a guide to identification”.^51^ The size of the colonies was verified by measuring the diameter in mm. Microscopic identification of filamentous fungi also occurred according to Larone et al.^51^ Through microscopic observation of the morphological structures, the hypha, conidium, conidiophore, phialide, foot cell, and vesicle were verified. Adhesive tape was used to transfer the colony to the slide, using cotton blue, and then visualization under a microscope was performed to identify the isolated filamentous fungal colonies to the genus level.

### 4.8 Statistical Analysis

The GraphPad Prism 8.0 software (GraphPad Inc., San Diego, USA) was used for statistical analysis. Data were subjected to analysis of variance (ANOVA), and differences between means were determined using Tukey’s test (*p* ≤ 0.05).

## Supplementary Material



## Notes

Figure 1S—Reduction reaction of resazurin to resofurin in the colorimetric method using the marker resazurin (7-hydroxy-3*H*-phenoxazine-3-one-10-oxide) (Merck, Darmstadt, Germany) to indicate the presence of viable cells; Table 1S—Macroscopic characteristics of fungi, identified according to Larone et al.,^51^ after 7 days of growth in SDA medium (Sabouraud dextrose agar) at 25 ± 3 °C, isolated from unsanitized strawberries, strawberries sanitized with sodium hypochlorite solution at 200 ppm and strawberries after application of essential oil (EO-PB), microemulsion (ME-PB) of *P. brevipedunculata* and white microemulsion (ME-WHITE); Table 2S—Microscopic characteristics of fungi identified according to Larone et al.,^51^ from fungi isolated from unsanitized strawberries, strawberries sanitized with a 200 ppm sodium hypochlorite solution, and strawberries after application of essential oil (EO-PB), microemulsion (ME-PB) of *P. brevipedunculata*, and white microemulsion (ME-WHITE); Figure 2S—Macroscopic characteristics (front and back) of colonies with 7 days of growth in acidified SDA medium (Sabouraud dextrose agar) at 25 ± 3 °C and 40× optical microscopy photograph with microscopic characteristics of the identified filamentous fungal genera; Table 3S—Presents different types of formulations, with various incorporated compounds, applied to strawberries with the aim of investigating various biological and chemical actions (PDF)
