# Supplementary material for: Antimicrobial Efficiency of Essential Oil and Microemulsion of Pectis brevipedunculata (Asteraceae): Evaluation of Bacterial and Fungal Inhibition in Strawberries
Source: ACS Omega. 2026 Apr 22;11(17):25596–614. doi: 10.1021/acsomega.6c00168 (PMC13150570; doi:10.1021/acsomega.6c00168)
Supplement: Supplementary file 1 [file ao6c00168_si_001.pdf]

Antimicrobial efficiency of essential oil and microemulsion of *Pectis brevipedunculata* (Asteraceae): Evaluation of bacterial and fungal inhibition in strawberries

Auxiliadora C. C. B. Lopes[1]; Roberto B. de Lima[1]; Renato S. Gonçalves[2]; Carlos Eduardo Lima de Oliveira[1]; Glécilla C. de S. Nunes[3]; Amanda M. Teles[4]; Matheus O. do Nascimento[5]; André L. M. Carvalho[5]; Cláudia Q. da Rocha[1*]

[1]Graduate Program in Chemistry, Federal University of Maranhão, São Luís 65080-805, MA, Brazil; rocha.claudia@ufma.br
[2]Department of Engineering and Exact Sciences, Setor Palotina, Federal University of Paraná, Palotina 85950-000, PR, Brazil
[3] Mechanical Engineering Department, State University of Maringá, Maringá 87020-900, PR, Brazil;
[4]Professional Postgraduate Program in Animal Health Defense, State University of Maranhão, São Luís 65055-310, MA, Brazil;
[5]Graduate Program in Pharmaceutical Sciences, Federal University of Piauí, Teresina 64049-550, PI, Brazil.

*Corresponding author:

rocha.claudia@ufma.br

ORCID: 0000-0002-3578-1869

Supporting Information

Figure 1S - Reduction of resazurin to resofurin in the presence of viable cells.

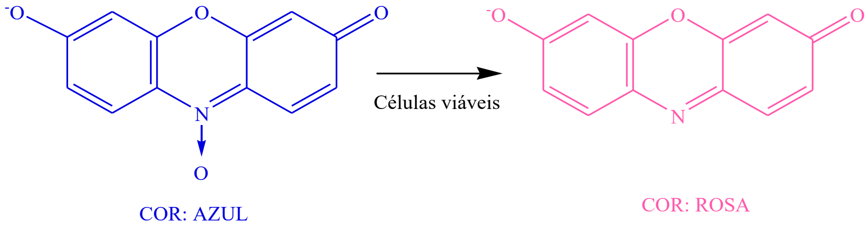

Source: Adapted from Chada[76].

Table 1S - Macroscopic characteristics of fungi after 7 days of growth in SDA (Sabouraud dextrose agar) medium at 25±3°C, isolated from unsanitized strawberries, strawberries sanitized with a 200 ppm sodium hypochlorite solution, and strawberries after application of essential oil (EO-PB), microemulsion (ME-PB) of *Pectis brevipedunculata*, and white microemulsion (ME-WHITE).

Fungus	Diameter (mm)	Texture	Pigmentation	Surface	Edge	Topography	Color	Appearance	Pigment	Growth
**1**	7.50	Velvety	Unchanged	Smooth and gray	Regular	Flat	Dark moss green	Dry	Absence	Fast
**2**	1.00	Suede	Unchanged	Cracked	Irregular and white	Pleated, umbilicated center	Green	Opaque	Absence	Slow
**3**	4.50	Powdery	Unchanged	Smooth	Irregular	Flat	Black	Moist	Absence	Fast
**4**	6.50	Leathery	Unchanged	Rough	Regular	Flat	Yellow, brown center	Shiny	Absence	Fast
**5**	3.00	Velvety	Unchanged	Cracked	Regular and white	Flat, pleated center	Green	Opaque	Absence	Fast
**6**	4.50	Suede	Unchanged	Cracked	Irregular and yellow	Pleated, umbilicated center	White	Opaque	Presence, yellow	Fast
**7**	2.30	Cotton-like	Unchanged	Smooth	Irregular	Umbilicated	White, purple center	Opaque	Absence	Fast
**8**	2.00	Suede with leathery edge	Changed	Smooth	Irregular and white	Umbilicated	Green, red center	Opaque	Presence, red	Fast
**9**	5.30	Powdery	Unchanged	Rough	Regular	Umbilicated	Black, white center	Opaque	Absence	Slow
**10**	3.00	Suede	Unchanged	Cracked	Irregular	Pleated, umbilicated center	White	Opaque	Absence	Slow

Source: Authors.

Table 2S - Microscopic characteristics of fungi isolated from unsanitized strawberries, strawberries sanitized with a 200 ppm sodium hypochlorite solution, and strawberries after application of essential oil (EO-PB), microemulsion (ME-PB) of *Pectis brevipedunculata*, and white microemulsion (ME-WHITE).

Fungus	Hypha	Conidium	Conidiophore	Fialide	Foot cell	Gallbladder
**1**	Septate and dark	Septate, dark	Short, septate, and dark	Absent	Present	Absent
**2**	Septate, dark	Stick, circular, dark			Present	
**3**	Absent	Round, smooth, dark	Long, dark	+ 7	Absent	Irradiated
**4**	Septate, hyaline	Round, hyaline	Short, septate	+ 7	Present	Flattened
**5**	Septate, hyaline	Round, smooth, hyaline	Short, septate, hyaline	3 a 5	Present	Absent
**6**	Septate, hyaline	Round, hyaline, smooth	Short/Long, septate, hyaline	3 a 4	Present	
**7**	Septate, hyaline	Circular, smooth, hyaline	Long	3 a 5	Present	Absent
**8**	Smooth, hyaline, septate	Smooth, spherical, hyaline	Short, smooth, hyaline	3 a 5	Present	Absent
**9**	Absent	Round, dark	Long, dark	Absent	Present	Irradiated
**10**	Septate, hyaline	Round, smooth, hyaline	Short, septate	3 a 4	Absent	Absent

Source: Authors.

Note: ND: Not determined.

Figure 2S - Macroscopic characteristics (reverse and obverse) of colonies with 7 days of growth in acidified SDA (Sabouraud dextrose agar) medium at 25±3°C and 40x optical microscopy photograph with microscopic characteristics of the identified filamentous fungi genera.

Fungus 1 - *Curvularia*

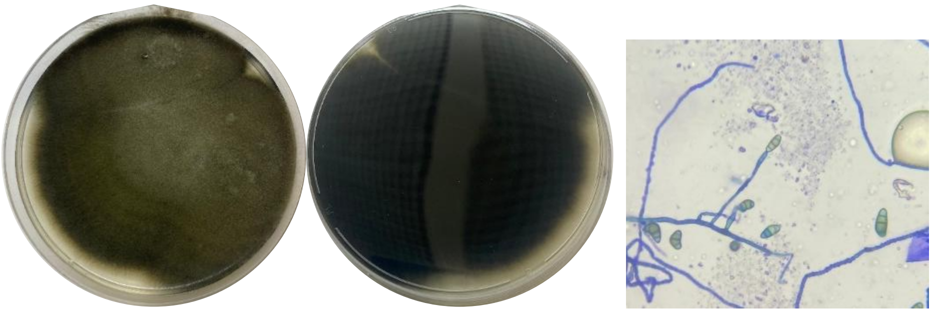

Fungus 2 - *Cladosporium*

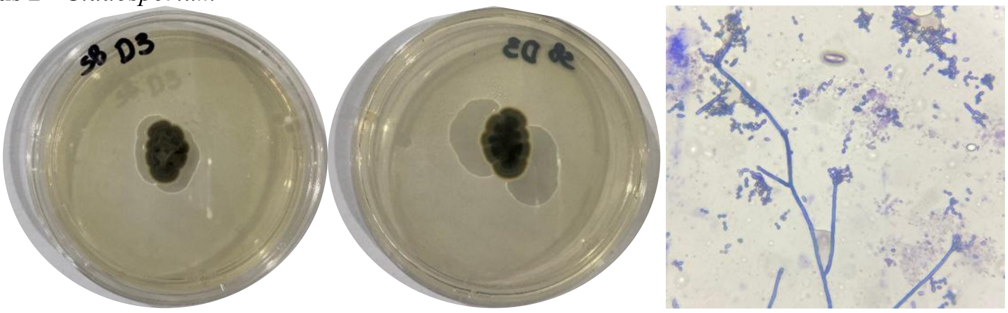

Fungus 3 - *Aspergillus*

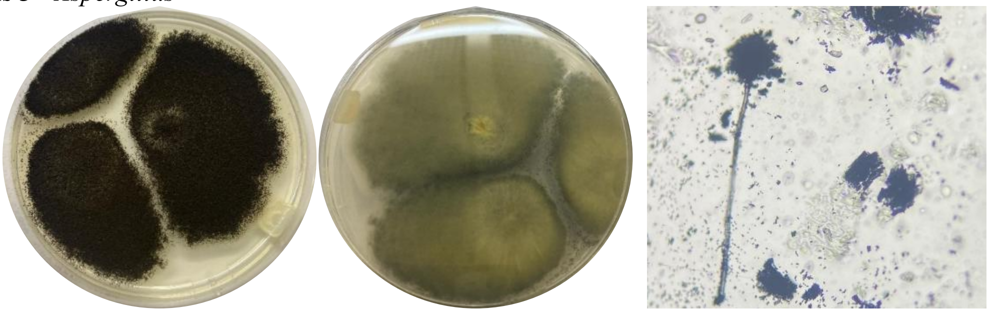

Fungus 4 – *Aspergillus*

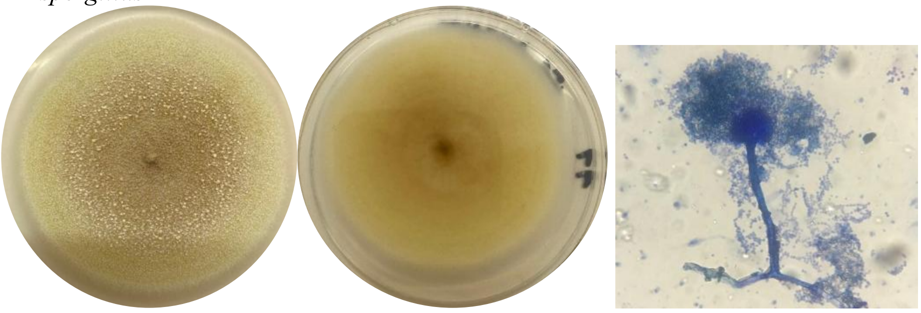

Fungus 5 – *Penicillium*

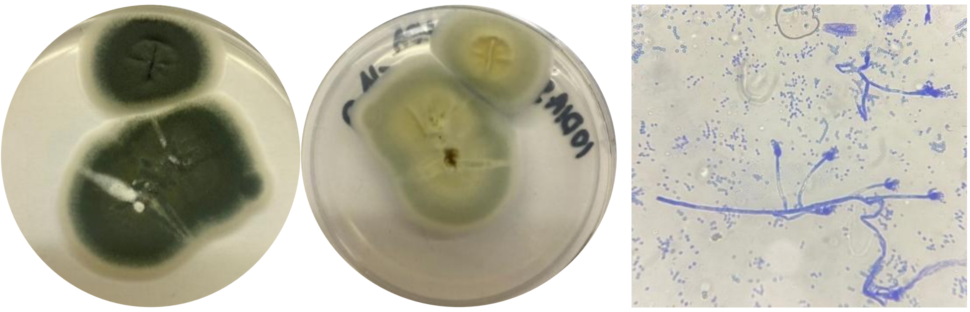

Fungus 6- *Penicillium*

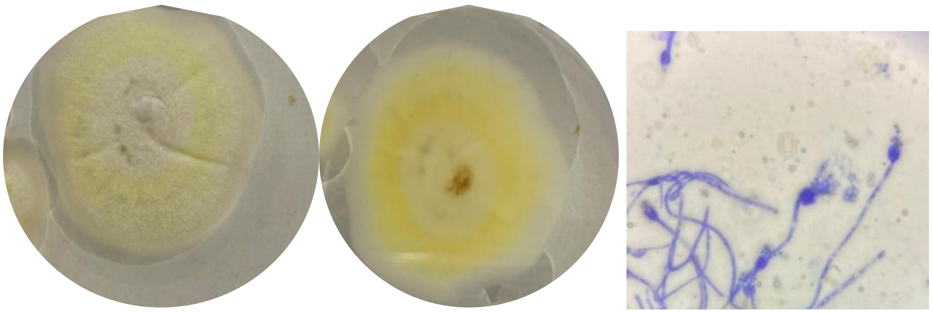

Fungus 7 – *Purpureocillium*

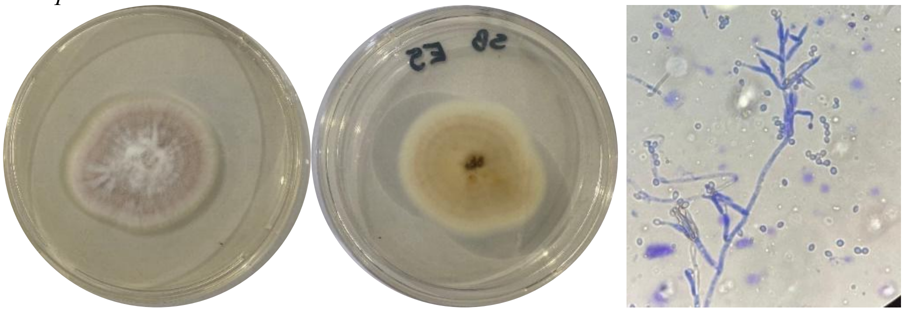

Fungus 8 - *Talaromyces*

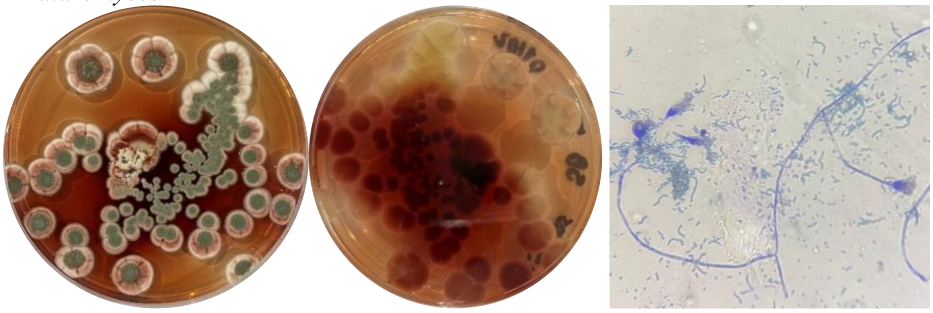

Fungus 9 - *Aspergillus*

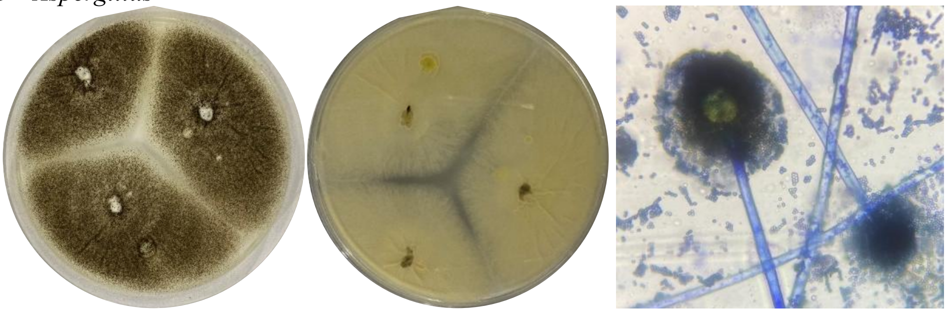

Fungus 10 - *Penicillium*

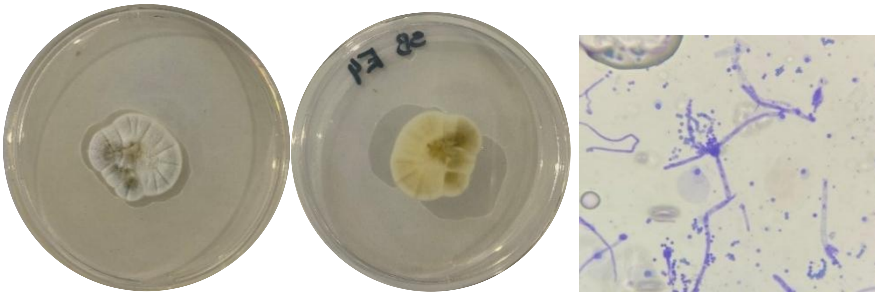

Source: Authors.

Table 3S – Different types of formulations, with various incorporated compounds, were applied to strawberries with the aim of investigating diverse biological and chemical actions.

FORMULATION TYPE	INCORPORATED COMPOUND	ACTION	FOOD	REFERENCES
Edible coating. Emulsion.	Chitosan, red thyme, oregano extract, limonene and peppermint	Antifungal activity	Strawberry	VU *et al*. (2011)[1]
Edible coating	Nanochitosan with and without copper	Antioxidant and antifungal activity	Strawberry	ESHGHI *et al*. (2014)[2]
Edible coating	Sodium alginate and pectin enriched with essential oil constituents (citral and eugenol)	Microbiological analysis	Strawberry	GUERREIRO *et al*. (2015)[3]
Nanoparticles	Chitosan and *Zataria multiflora* essential oil	Antifungal activity	Strawberry	MOHAMMADI *et al*. (2015)[4]
Edible coating	Chitosan with nisin, natamycin, pomegranate and grape seed extract.	Microbiological analysis	Strawberry	DURAN *et al*. (2016)[5]
Edible coating	Lemon essential oil and chitosan	Characterization of the maturity index of the respiration rate, and volatile analysis	Strawberry	PERDONES *et al*. (2016)[6]
Edible coating	Alginate with carvacrol and methyl cinnamate	Microbiological analysis	Strawberry	PERETTO *et al*. (2017)[7]
Edible coating	Chitosan with monomethylfumaric acid	Microbiological analysis	Strawberry	KHAN *et al*. (2019)[8]
Nanoemulsion	Chitosan and nutmeg seed oil	Microbiological analysis	Strawberry	HORISON *et al*. (2019)[9]
Edible coating	Chitosan, sodium alginate and oregano essential oil	Antifungal activity	Strawberry	LEE *et al*. (2022)[10]
Edible coating film composed of microemulsion	Chitosan/silk fibroin with a microemulsion of cinnamon essential oil	Antibacterial activity	Strawberry	CHEN *et al*. (2024)[11]
Nanoemulsion	Rosemary essential oil (*Rosmarinus officinalis*)	Antifungal activity	Strawberry	RAMOS *et al*. (2024)[12]

Formulation with gums	Gum arabic, guar gum, citric acid, tartaric acid, and malic acid.	Microbiological analysis	Strawberry	CHOUDHARY *et al.* (2025) [13]
Nanoemulsion	*Baccharis dracunculifolia* essential oil and nerolidol	Antifungal activity and antioxidant	Strawberry	CORRÊA *et al.* (2025) [14]
Nanoemulsion	*Satureja hortensis* essential oil	Evaluation of physicochemical parameters and sensory attributes (color, texture, flavor, aroma, and overall acceptability)	Strawberry	HASSANI *et al.* (2025) [15]
Nanoemulsion	*Green cinnamon* essential oil	Antimicrobial activity	Strawberry	ZHU *et al.* (2025) [16]
Nanoemulsion	Chitosan with essential oil of *Ocimum gratissimum* L.	Antifungal activity	Strawberry	SONI *et al.* (2026) [17]
Nanostructured films	Starch with essential oil of *Baccharis dracunculifolia* and nerolidol	Antifungal activity	Strawberry	CORRÊA *et al.* (2026) [18]
Gel film with Nanoemulsion	Clove essential oil	Antibacterial and antioxidant activity	Strawberry	LIU *et al.* (2026) [19]
Edible film	Essential oil of *Melaleuca bracteata* F. Muell, chitosan and gelatin	Antimicrobial and antioxidant activity	Strawberry	YANG *et al.* (2026) [20]

Source: Authors.

REFERENCES

(1) VU, K.D.; HOLLINGSWORTH, R.G. ; LEROUX, E.; SALMIERI, S.; LACROIX. M. Development of edible bioactive coating based on modified chitosan for increasing the shelf life of strawberries. **Food Research International** 44 (2011) 198–203. doi:10.1016/j.foodres.2010.10.037

(2) ESHGHI, S.; HASHEMI, M.; MOHAMMADI, A.; BADII, F.; MOHAMMADHOSEINI, Z.; AHMADI, K. Effect of Nanochitosan-Based Coating With and Without Copper Loaded on Physicochemical and Bioactive Components of Fresh Strawberry Fruit (*Fragaria* x *ananassa Duchesne*) During Storage. **Food Bioprocess Technol** (2014) 7:2397–2409 DOI 10.1007/s11947-014-1281-2

(3) GUERREIRO, A. C.; GAGO, C. M. L.; FALEIRO, M. L.; MIGUEL, M. G.C.; ANTUNES, M. D.C. The use of polysaccharide-based edible coatings enriched with essential oils to improve shelf-life of strawberries. **Postharvest Biology and Technology**, v.110, p.51-60, 2015. https://doi.org/10.1016/j.postharvbio.2015.06.019.

(4) MOHAMMADI, A.; HASHEMI, M.; HOSSEINI, S. M. Nanoencapsulation of *Zataria multiflora* essential oil preparation and characterization with enhanced antifungal activity for controlling *Botrytis cinerea*, the causal agent of gray mould disease, **Innovative Food Science & Emerging Technologies**, v. 28, 2015, p. 73-80, https://doi.org/10.1016/j.ifset.2014.12.011.

(5) DURAN, M.; ADAY, M. S.; ZORBA, N. N. D.; TEMIZKAN, R.; BÜYÜKCAN, M. B.; CANER, C. Potential of antimicrobial active packaging 'containing natamycin, nisin, pomegranate and grape seed extract in chitosan coating' to extend shelf life of fresh strawberry. **Food and Bioproducts Processing**, v.98, 2016, p.354-363, https://doi.org/10.1016/j.fbp.2016.01.007.

(6) PERDONES, A.; ESCRICHE, I.; CHIRALT, A.; VARGAS, M. Effect of chitosan–lemon essential oil coatings on volatile profile of strawberries during storage, **Food Chemistry**, v. 197, 2016, p. 979-986, https://doi.org/10.1016/j.foodchem.2015.11.054.

(7) PERETTO, G.; DU, W.; AVENA-BUSTILLOS, R. J.; BERRIOS, J. J.; SAMBO, P.; MCHUGH, T. H. Electrostatic and Conventional Spraying of Alginate-Based Edible Coating with Natural Antimicrobials for Preserving Fresh Strawberry Quality. **Food Bioprocess Technol** (2017) 10:165–174. DOI 10.1007/s11947-016-1808-9

(8) KHAN, I.; TANGO, C. N.; CHELLIAH, R.; OH, D. H. Development of antimicrobial edible coating based on modified chitosan for the improvement of strawberries shelf life. **Food Science and Biotechnology**, v.28, n.4, p.1257–1264, 2019. https://doi.org/10.1007/s10068-018-00554-9

(9) HORISON, R.; SULAIMAN, F.O.; ALFREDO, D.; WARDANA, A. A. Physical characteristics of nanoemulsion from chitosan/nutmeg seed oil and evaluation of its coating against microbial growth on strawberry. **Food Research**, v.3, n.6, p.821 – 827, 2019. https://doi.org/10.26656/fr.2017.3(6).159

(10) LEE, D.; SHAYAN, M.; GWON, J.; PICHA, D. H.; WU, Q. Effectiveness of cellulose and chitosan nanomaterial coatings with essential oil on postharvest strawberry quality, **Carbohydrate Polymers**, v. 298, 2022, 120101, https://doi.org/10.1016/j.carbpol.2022.120101.

(11) CHEN, X.; HU, X.; ZHANG, J. Properties of an active film based on chitosan/silk fibroin loaded with an essential oil microemulsion and its application in preservation of strawberries, **Food Packaging and Shelf Life**, v. 43, 2024, 101270, https://doi.org/10.1016/j.fpsl.2024.101270.

(12) RAMOS, E. G.; QUEIROZ, A. G.; VELEIRINHO, M. B. R.; FELIPINI, R. B.; DI PIERO, R. M. Nanoformulations containing rosemary oil for gray mold control in strawberries, **Scientia Horticulturae**, v. 338, 2024, 113678, https://doi.org/10.1016/j.scienta.2024.113678.

(13) CHOUDHARY, A.; TOSIF, M. M.; BAINS, A.; GOKSEN, G.; NAGRAIK, R.; DHULL, S. B.; ALI, N.; MUZAFFAR, N.; CHAWLA, P. Impact of organic acid cross-linking on the structure and functional properties of gum arabic and guar gum: Formulation of an edible coating for enhancing strawberry shelf life, **Food Chemistry**: X, v. 28, 2025, 102527, https://doi.org/10.1016/j.fochx.2025.102527.

(14) CORRÊA, A. N. R.; CLERICI, N. J.; VENCATO, A. A.; BRANDELLI, A. Nano-coatings with *Baccharis dracunculifolia* essential oil and nerolidol as an antifungal and antioxidant strategy in post-harvest strawberries, **Biocatalysis and Agricultural Biotechnology**, v. 70, 2025, 103830,https://doi.org/10.1016/j.bcab.2025.103830.

(15) HASSANI, B.; EBRAHIMI, F.; NAJAFI, A.; ZIAOLHAGH, S. Investigation of the effect of aloe vera gel coating combined with free and encapsulated savory essential oil on the shelf life of strawberries, **Applied Food Research**, v. 5, Issue 2, 2025, 101269, https://doi.org/10.1016/j.afres.2025.101269.

(16) ZHU, Y.; CHEN, T.; MENG, Z.; LI, T.; ZHANG, J.; ZHANG, N.; LUO, G.; WANG, Z.; ZHOU, Y. Preparation and characterization of a novel green cinnamon essential oil nanoemulsion for the enhancement of safety and shelf-life of strawberries, International **Journal of Food Microbiology**, v. 427, 2025, 110935, https://doi.org/10.1016/j.ijfoodmicro.2024.110935.

(17) SONI, M.; PAUL, K. K.; YADAV, M.; AGNIHOTRI, P.; TIWARI, P.; DWIVEDY, A. K. Enhancing the shelf-life of strawberry using chitosan-based edible coating of *Ocimum gratissimum* L. essential oil nanoemulsion, **Plant Nano Biology**, v. 15, 2026, 100238, https://doi.org/10.1016/j.plana.2025.100238.

(18) CORRÊA, A. N. R.; CRIZEL, T. M.; ZANOTELLI, L. E.; FLORES, S. H.; BRANDELLI, A. Nanostructured starch-based films incorporating essential oil and nerolidol-loaded nanoparticles for postharvest protection of strawberries, **International Journal of Biological Macromolecules**, v. 340, Part 2, 2026, 150168, https://doi.org/10.1016/j.ijbiomac.2026.150168.

(19) LIU, Y.; LIU, Z.; WANG, Y.; MA, Y.; ZHANG, L.; LIU, M.; WANG, S. Biocompatible κ-carrageenan gel films with clove essential oil nanoemulsion: Optimization, properties, and strawberry preservation efficacy, **Food Hydrocolloids**, v. 174, 2026, 112392, https://doi.org/10.1016/j.foodhyd.2025.112392

(20) YANG, J.; NIU, Y.; HU, P.; SONG, Y.; DANG, R.; LI, L.; ZHANG, Y.; GONG, Y.; LIN, Y.; XIN, Y. Biopolymer-based edible film incorporating *Melaleuca Bracteata* F. Muell. Essential oil with antimicrobial and antioxidant properties: A promising approach for strawberry fruit preservation, **Food Research International**, v. 229, 2026, 118498, https://doi.org/10.1016/j.foodres.2026.118498.
